# Synthesis and biological evaluation of thiazolidine-2-thione derivatives as novel xanthine oxidase inhibitors

**DOI:** 10.1371/journal.pone.0268531

**Published:** 2022-05-18

**Authors:** Mu-Xuan Wang, Hong-Wei Qin, Chao Liu, Shen-Ming Lv, Jia-Shu Chen, Chun-Gu Wang, Ying-Ying Chen, Jia-Wei Wang, Jin-Yue Sun, Zhi-Xin Liao

**Affiliations:** 1 Department of Pharmaceutical Engineering, School of Chemistry and Chemical Engineering and Jiangsu Province Hi-Tech Key Laboratory for Biomedical Research, Southeast University, Nanjing, Jiangsu, P.R. China; 2 School of Life Sciences and Bioengineering, Jining University, Qufu, Shandong, P.R. China; 3 Key Laboratory of Novel Food Resources Processing, Ministry of Agriculture and Rural Affairs/Key Laboratory of Agro-Products Processing Technology of Shandong Province/Institute of Agro-Food Science and Technology, Shandong Academy of Agricultural Sciences, Jinan, Shandong, P.R. China; Institute of Molecular Genetics of Czech Academy of Sciences: Ustav molekularni genetiky Akademie Ved Ceske Republiky, CZECH REPUBLIC

## Abstract

Xanthine oxidase (XO) is a key enzyme in the generation and development of hyperuricemia. Thiazolidine-2-thione, a typical heterocyclic compound, have been widely used in the field of drug synthesis. In this study, a series of novel thiazolidine-2-thione derivatives were synthesized as XO inhibitors, and the XO inhibitory potencies of obtained compounds were evaluated by *in vitro* enzyme catalysis. The result shown that compound **6k** behaved the strongest XO inhibitory activity with an IC_50_ value of 3.56 μmol/L, which was approximately 2.5-fold more potent than allopurinol. The structure-activity relationship revealed that the phenyl-sulfonamide group was indispensable for thiazolidine-2-thione derivatives to produce XO inhibitory activity. The enzyme inhibition kinetics analyses confirmed that compound **6k** exerted a mixed-type XO inhibition. Additionally, the molecular docking results suggested that the 4-fluorophenyl-sulfonyl moiety could interact with Gly260 and Ile264 in the innermost part of the active pocket through 2 hydrogen bonds, while the thiazolidinethione moiety could form two hydrogen bonds with Glu263 and Ser347 in hydrophobic pockets. In summary, the results described above suggested that compound **6k** could be a valuable lead compound for the treatment of hyperuricemia as a novel XO inhibitor.

## 1. Introduction

Xanthine oxidase (XO) is a key enzyme in purine catabolism in some species including humans, which plays a major role in the oxidation of hypoxanthine to xanthine, and then xanthine to uric acid (UA) [1, 2]. Overproduction of uric acid induces hyperuricemia due to abnormal XO activity, which is also linked with gout. Gout is a metabolic disease in which excessive levels of UA cause deposition of urate crystals in joints [3–5]. In addition, hyperuricemia is also associated with other diseases, such as inflammation [6], chronic kidney disease [7, 8], hypertensive disorders [9] and cardiovascular diseases [10]. XO inhibitors can block the biosynthesis of UA from purines, which can lower the production of UA. Allopurinol [11], febuxostat [9] and topiroxostat [12] are the clinical inhibitors of XO, used for the treatment of hyperuricemia. Current inhibitors of XO have several adverse effects (e.g. skin rashes, allergic reactions, increased blood pressure and increased risk of developing cataracts) [13–15], so there is a need of new XO inhibitors with better efficacy, and lower side effects.

Due to the structural characteristics and superior biological activity, heterocyclic compounds have a wide range of applications in the field of medicinal synthesis. Recently, research emphasis has switched to the discovery of novel effective and affordable XO inhibitors with minimal side effects based on heterocyclic compounds [16–18]. Thiazolidines are the representatives of five-membered heterocyclic compound, which have been used in different areas of medicine [19, 20], materials [21], biological dyes [22], and ion receptors [23]. As a typical thiazolidine derivatives, thiazolidine-2-thione is an important organic intermediate in pharmaceuticals and agrochemicals. Thiazolidine-2-thione show diversified biological activity, such as aldose reductase inhibitors, anticancer, anti-inflammatory, and antifungal [24, 25]. At the same time, thiazolidine-2-thione has two tautomers [26, 27], including thione and thiol, which also has been widely used as chiral auxiliaries in catalytic asymmetric synthesis.

In this study, a series of novel thiazolidine-2-thione derivatives were designed and synthesized. *In vitro* XO inhibitory activity was researched using enzyme catalysis, and structure-activity relationships of thiazolidine-2-thione derivatives were described. The inhibitory mode of compound **6k** was determined through enzyme inhibitory kinetics studies. In addition, molecular modeling study was also performed to investigate the inhibitory behaviors of compound **6k**.

## 2. Materials and methods

### 2.1 Chemistry synthesis

The chemicals for the synthetic reactions were purchased from Macklin Biochemical Co. Ltd, and other organic reagents were purchased from local reagent dealers. Melting points (m.p.) were obtained with MP-21 micro melting point apparatus. ^1^H and ^13^C NMR spectra were performed on a Bruker AV-400 nuclear magnetic resonance for solutions of the compounds in CDCl_3_ at a temperature of 23–28°C, *J* values are given in Hz. High-resolution mass spectra were recorded on an Agilent 6520 Series Q-TOF-MS system. All reactions were monitored by thin-layer chromatography (TLC).

#### 2.1.1 Preparation of 2-aminoethanol hydrogen sulfate (2)

Aminoethanol (9 mL, 0.15 mol) and H_2_O (9 mL) were added 100 mL three-necked flask respectively at 0°C, and then, the mixture (16.3 mL, v/v = 1/1) of sulfuric acid (98%) and H_2_O was slowly dropped into the flask. The reaction mixture was stirred at 0°C for 0.5 h. After the completion of the reaction, the mixture was added to absolute ethanol, filtered, and the filter cake was washed with absolute ethanol (3×15 mL). Drying under vacuum gave 2-aminoethanol hydrogen sulfate (19.2 g, yield 90.7%) and can be applied in next step without further purification.

*2-aminoethanol hydrogen sulfate* (**2**). white solid, m.p. 74–75°C. ESI-MS: m/z calc. for C_2_H_7_NO_4_S 142.0175 [M+H]^+^, found 142.0207 [M+H]^+^.

#### 2.1.2 Preparation of thiazolidine-2-thione (3)

Intermediate **2** (7.06 g, 0.05 mol), KOH (5.61 g, 0.10 mol) and ethanol (100 mL) were added 100 mL three-necked flask respectively. The reaction mixture was heated to 40°C and carbon disulfide (7.61 g, 0.10 mol) were added in ten batches about 1 hour. The reaction mixture was further stirred at that temperature for 3 h. After the completion of the reaction, the mixture was allowed to cool to 5~10°C and washed with 5% sodium hydroxide solution (100 mL). The extract was washed with saturated brine (3×10 mL), dried over anhydrous sodium sulfate and concentrated under reduced pressure to obtain the crude. Dissolve the crude in absolute ethanol for recrystallization to obtain pure thiazolidine-2-thione (4.05 g, yield 68.1%).

*thiazolidine-2-thione* (**3**). white solid, m.p.: 105–107°C, ESI-MS: m/z calc. for C_3_H_5_NS_2_ 119.9942 [M+H]^+^, found 119.9913 [M+H]^+^.

#### 2.1.3 General procedure for the preparation of compound 4a-4d

Thiazolidine-2-thione (1.20 g, 10.0 mmol), NaOH (0.44 g, 11.0 mmol), and ethanol (40 mL) were added 100 mL three-necked flask respectively at 40°C. After NaOH was completely dissolved, a mixed solution of bromoethane/bromopropane (15.0 mmol) and ethanol (15 mL) was slowly added dropwise using a constant pressure funnel. Then the temperature was raised to 50°C and the reaction was carried out for 5 h. The progress of the reaction was monitored by TLC. After the completion of the reaction, the mixture was allowed to cool to room temperature and extracted with ethyl acetate (3×10 mL). The extract was washed with saturated brine (3×10 mL), dried over anhydrous sodium sulfate and concentrated under reduced pressure. The residue was purified by column chromatography eluting with a mixture of petroleum ether and ethyl acetate (10:1–1:1).

*3-ethyl-thiazolidine-2-thione* (**4a**). Colorless oily liquid (1.06 g, yield 71.9%). ^1^H-NMR *δ*_*H*_ (400 MHz, СDCl_3_): 4.21 (t, *J* = 7.8 Hz, 2H), 3.38 (q, 2H), 3.10 (t, *J* = 7.8 Hz, 2H), 1.36 (t, *J* = 7.2 Hz, 3H). ^13^C-NMR *δ*_*C*_ (100 MHz, СDCl_3_): 163.44, 64.37, 35.23, 27.09, 14.69. ESI-MS: m/z calc. for C_5_H_9_NS_2_ 148.0855 [M+H]^+^, found 148.0801 [M+H]^+^.

*3-propyl-thiazolidine-2-thione* (**4b**). Colorless oily liquid. (1.01 g, yield 68.3%). ^1^H-NMR *δ*_*H*_ (400 MHz, CDCl_3_): 4.07 (t, *J* = 6.0 Hz, 2H), 3.75 (t, *J* = 6.0 Hz, 2H), 3.27 (t, *J* = 9.0 Hz, 2H), 1.65 (m, 2H), 0.87 (t, *J* = 6.0 Hz, 3H). ^13^C-NMR *δ*_*C*_ (100 MHz, СDCl_3_): 164.75, 67.14, 59.62, 38.31, 27.22, 14.58. ESI-MS: m/z calc. for C_6_H_11_NS_2_ 162.0412 [M+H]^+^, found 162.0433 [M+H]^+^.

Thiazolidine-2-thione (1.20 g, 10.0 mmol), NaOH (0.44 g, 11.0 mmol), CuI (0.1g, 0.5 mmol) and ethanol (40 mL) were added 100 mL three-necked flask respectively at 60°C. After NaOH was completely dissolved, a mixed solution of bromobenzene/benzyl bromide (15.0 mmol) and ethanol (15 mL) was slowly added dropwise using a constant pressure funnel. Then the temperature was raised to 80°C and the reaction was carried out for 16 h. The progress of the reaction was monitored by TLC. After the completion of the reaction, the mixture was allowed to cool to room temperature and extracted with ethyl acetate (3×10 mL). The extract was washed with saturated brine (3×10 mL), dried over anhydrous sodium sulfate and concentrated under reduced pressure. The residue was purified by column chromatography eluting with a mixture of petroleum ether and ethyl acetate (10:1–1:1).

*3-phenyl-thiazolidine-2-thione* (**4c**). Colorless oily liquid. (1.28 g, yield 66.7%). ^1^H-NMR *δ*_*H*_ (400 MHz, CDCl_3_): 7.72 (d, *J* = 13.1 Hz, 2H), 7.33 (t, *J* = 10.4 Hz, 2H), 7.09 (t, *J* = 8.3 Hz, 2H), 3.81 (t, *J* = 5.4 Hz, 2H), 3.27 (t, *J* = 14.3 Hz, 2H). ^13^C-NMR *δ*_*C*_ (100 MHz, СDCl_3_): 191.34, 152.27, 137.51, 130.44, 128.83, 44.19, 35.72. ESI-MS: m/z calc. for C_9_H_9_NS_2_ 196.0255 [M+H]^+^, found 196.0207 [M+H]^+^.

*3-benzyl-thiazolidine-2-thione* (**4d**). White powder solid (1.44 g, yield 68.9%), mp: 115–117°C. ^1^H-NMR *δ*_*H*_ (400MHz, СDCl_3_): 7.34 (m, 5H), 4.42 (d, 2H), 4.25 (t, J = 9.0 Hz, 2H), 3.41 (t, J = 9.0 Hz, 2H). ^13^C-NMR *δ*_*C*_ (100 MHz, СDCl_3_): 197.15, 138.82, 128.94, 126.52, 124.43, 57.27, 44.92, 23.61. ESI-MS: m/z calc. for C_10_H_11_NS_2_ 210.0412 [M+H]^+^, found 210.0405 [M+H]^+^.

#### 2.1.4 Preparation of 2-thioxothiazolidine-3-carbonyl chloride (5)

Thiazolidine-2-thione (1.20 g, 10.0 mmol), triethylamine (TEA, 1.34 g, 13.2 mmol), and CH_2_Cl_2_ (80 mL) were added 250 mL three-necked flask respectively and stirred at 0°C. Triphosgene (1.32 g, 4.4 mmol) was dissolved in CH_2_Cl_2_ (20 mL) and slowly added dropwise to three-necked flask using a constant pressure funnel. Then, the reaction mixture was stirred overnight at room temperature. After completion of the reaction as indicated by TLC, the mixture was poured to pre-cooled 1mol/L HCl (60 mL), and the product was extracted with CH_2_Cl_2_ (3×10 mL). The combined organic phase was washed successively with saturated sodium bicarbonate and sodium chloride, dried over sodium sulfate, and concentrated *in vacuo*. The solid was recrystallized with petroleum ether to obtain yellow crystals 2-thioxothiazolidine-3-carbonyl chloride (1.46 g, yield 80.37%), m.p. 88–90°C.

#### 2.1.5 General procedure for the preparation of compound 6a-6k

Amine (10 mmol), TEA (0.67g, 6.6 mmol) and tetrahydrofuran (THF, 40 mL) were added 100 mL three-necked flask respectively and stirred at 0°C. Compound **5** (1.10 g, 6.0 mmol) was dissolved in THF (10 mL) and slowly added dropwise to three-necked flask using a constant pressure funnel. Then, the reaction mixture was stirred for 8 h at room temperature and monitored by TLC. After completion of the reaction, the mixture was extracted with CH_2_Cl_2_ (3×25 mL), and the combined organic phase was washed successively with saturated sodium chloride, dried over anhydrous sodium sulfate, and concentrated *in vacuo*. The residue was purified by through column chromatography (petroleum ether/ethyl acetate) to obtain the desired compound **6a-6k**.

*N-ethyl-2-thioxothiazolidine-3-carboxamide* (**6a**). Light yellow oil liquid (1.18 g, yield 90.08%). ^1^H-NMR *δ*_*H*_ (400 MHz, CDCl_3_): 9.89 (s, 1H), 4.76 (t, *J* = 6.0 Hz, 2H), 3.99 (t, *J* = 9.0 Hz, 2H), 3.26 (t, *J* = 7.8 Hz, 2H), 1.15 (t, *J* = 5.4 Hz, 3H). ^13^C-NMR *δ*_*C*_ (100 MHz, СDCl_3_): 200.22, 151.36, 56.27, 43.35, 26.99, 22.51. ESI-MS: m/z calc. for C_6_H_10_N_2_OS_2_ 213.0235 [M+Na]^+^, found 213.0234 [M+Na]^+^.

*N-propyl-2-thioxothiazolidine-3-carboxamide* (**6b**). Light yellow oil liquid (0.94 g, yield 76.68%). ^1^H-NMR *δ*_*H*_ (400 MHz, CDCl_3_): 9.80 (s, 1H), 4.78 (t, *J* = 6.0 Hz, 2H), 3.31–3.25 (m, 4H), 1.54–1.66 (m, 2H), 0.96 (t, *J* = 6.0 Hz, 3H). ^13^C-NMR *δ*_*C*_ (100 MHz, СDCl_3_): 200.28, 152.30, 56.35, 42.40, 27.10, 22.42, 11.55. ESI-MS: m/z calc. for C_7_H_12_N_2_OS_2_ 227.0283 [M+Na]^+^, found 227.0282 [M+Na]^+^.

*N-butyl-2-thioxothiazolidine-3-carboxamide* (**6c**). Light yellow oil liquid (1.02 g, yield 77.86%). ^1^H-NMR *δ*_*H*_ (400 MHz, CDCl_3_): 9.97 (s, 1H), 4.89 (m, 2H), 3.82 (m, 2H), 3.53 (m, 2H), 1.66 (m, 2H), 1.23 (t, *J* = 6.0 Hz, 2H). ^13^C-NMR *δ*_*C*_ (100 MHz, СDCl_3_): 200.16, 152.32, 56.30, 40.38, 31.15, 27.06, 20.12, 13.65. ESI-MS: m/z calc. for C_8_H_14_N_2_OS_2_ 241.0439 [M+Na]^+^, found 241.0420 [M+Na]^+^.

*N-isobutyl-2-thioxothiazolidine-3-carboxamide* (**6d**). Light yellow oil liquid (0.92 g, yield 70.23%). ^1^H-NMR *δ*_*H*_ (400 MHz, CDCl_3_): 9.84 (s, 1H), 4.70 (t, *J* = 9.0 Hz, 2H), 3.28 (t, *J* = 7.8 Hz, 2H), 3.16 (t, *J* = 5.7 Hz, 2H), 1.86 (m, 1H), 0.95 (d, *J* = 6.0 Hz, 6H). ^13^C-NMR *δ*_*C*_ (100 MHz, СDCl_3_): 200.32, 152.43, 56.36, 48.19, 28.13, 27.09, 20.22. ESI-MS: m/z calc. for C_8_H_14_N_2_OS_2_ 241.0439 [M+Na]^+^, found 241.0429 [M+Na]^+^.

*N-phenyl-2-thioxothiazolidine-3-carboxamide* (**6e**). Yellow solid powder (2.15 g, yield 89.84%), m.p. 101–102°C. ^1^H-NMR *δ*_*H*_ (400 MHz, CDCl_3_): 8.17 (s, 1H), 7.44–7.38 (m, 2H), 7.31–7.18 (m, 2H), 4.69 (t, *J* = 5.3 Hz, 2H), 3.40 (t, *J* = 9.0 Hz, 2H). ^13^C-NMR *δ*_*C*_ (100 MHz, СDCl_3_): 201.83, 150.17, 149.67, 129.55, 126.45, 121.26, 55.83, 28.43. ESI-MS: m/z calc. for C_10_H_10_N_2_OS_2_ 261.0235 [M+Na]^+^, found 261.0229 [M+Na]^+^.

*N-benzyl-2-thioxothiazolidine-3-carboxamide* (**6f**). Yellow solid powder (0.98 g, yield 64.72%), m.p. 107–109°C. ^1^H-NMR *δ*_*H*_ (400 MHz, CDCl_3_): 10.25 (s, 1H), 7.37–7.24 (m, 5H), 4.70 (t, *J* = 8.3 Hz, 2H), 4.50 (t, *J* = 6.7 Hz, 2H), 3.24 (t, *J* = 9.0 Hz, 2H). ^13^C-NMR *δ*_*C*_ (100 MHz, СDCl_3_): 200.55, 152.48, 137.49, 128.70, 127.57, 127.53, 56.34, 44.61, 27.13. ESI-MS: m/z calc. for C_11_H_12_N_2_OS_2_ 275.0391 [M+Na]^+^, found 275.0328 [M+Na]^+^.

*N-(pyridin-3-yl)-2-thioxothiazolidine-3-carboxamide* (**6g**). Yellow solid powder (0.98 g, yield 64.72%), m.p. 111–113°C. ^1^H-NMR *δ*_*H*_ (400 MHz, CDCl_3_): 9.60 (s, 1H), 7.87 (m, 1H),

7.73 (m, 1H), 7.51 (m, 1H), 7.24 (t, *J* = 7.2 Hz, 1H), 3.56 (t, *J* = 6.3 Hz, 2H), 3.18 (t, *J* = 9.4 Hz, 2H). ^13^C-NMR *δ*_*C*_ (100 MHz, СDCl_3_): 200.26, 152.32, 141.43, 140.16, 138.27, 121.94, 119.73, 58.30, 21.96. ESI-MS: m/z calc. for C_9_H_9_N_3_OS_2_ 262.0715 [M+Na]^+^, found 262.0817 [M+Na]^+^.

*N-cyclohexyl-2-thioxothiazolidine-3-carboxamide* (**6h**). White solid powder (1.10 g, yield 75.02%), m.p. 114–115°C. ^1^H-NMR *δ*_*H*_ (400 MHz, CDCl_3_): 7.46 (s, 1H), 4.53 (t, *J* = 11.4 Hz, 2H), 4.26 (m, 2H), 2.45–2.11 (t, *J* = 7.8 Hz, 2H), 1.75–1.28 (m, 4H), 0.95–0.86 (m, 6H). ^13^C-NMR *δ*_*C*_ (100 MHz, СDCl_3_): 200.26, 151.42, 56.28, 42.68, 32.38, 26.99, 25.53, 18.23. ESI-MS: m/z calc. for C_10_H_16_N_2_OS_2_ 267.0596 [M+Na]^+^, found 267.0531 [M+Na]^+^.

*N-(phenylsulfonyl)-2-thioxothiazolidine-3-carboxamide* (**6i**). White solid powder (1.57 g, yield 77.48%), m.p. 121–123°C. ^1^H-NMR *δ*_*H*_ (400 MHz, CDCl_3_): 9.87 (s, 1H), 7.86 (t, *J* = 15.2 Hz, 3H), 7.46 (m, 2H), 3.48 (t, *J* = 9.4 Hz, 2H), 3.42 (t, *J* = 9.0 Hz, 2H). ^13^C-NMR *δ*_*C*_ (100 MHz, СDCl_3_): 201.27, 153.47, 134.49, 132.53, 120.04, 56.41, 33.16. ESI-MS: m/z calc. for C_10_H_10_N_2_O_3_S_3_ 324.9752 [M+Na]^+^, found 324.9803 [M+Na]^+^.

*N-((4-methylphenyl)sulfonyl)-2-thioxothiazolidine-3-carboxamide* (**6j**). White solid powder (0.90 g, yield 66.58%), m.p. 127–129°C. ^1^H-NMR *δ*_*H*_ (400 MHz, CDCl_3_): 9.54 (s, 1H), 7.54 (d, *J* = 13.2 Hz, 2H), 7.29 (d, *J* = 7.2 Hz, 2H), 3.56(t, *J* = 8.3 Hz, 2H), 3.47 (t, *J* = 11.2 Hz, 2H), 1.25 (m, 3H). ^13^C-NMR *δ*_*C*_ (100 MHz, СDCl_3_): 163.73, 145.81, 137.62, 131.44, 128.36, 33.21, 23.48, 19.32. ESI-MS: m/z calc. for C_11_H_12_N_2_O_3_S_3_ 338.9908 [M+Na]^+^, found 338.9874 [M+Na]^+^.

*N-((4-fluorophenyl)sulfonyl)-2-thioxothiazolidine-3-carboxamide* (**6k**). White solid powder (1.01 g, yield 74.72%), m.p. 128–129°C. ^1^H-NMR *δ*_*H*_ (400 MHz, CDCl_3_): 9.97 (s, 1H), 7.64 (t, *J* = 13.5 Hz, 2H), 7.45 (t, *J* = 9.6 Hz, 2H), 4.93 (t, *J* = 6.8 Hz, 2H), 3.23 (t, *J* = 8.4 Hz, 2H). ^13^C-NMR *δ*_*C*_ (100 MHz, СDCl_3_): 201.16, 153.43, 138.31, 129.94, 129.62, 57.72, 45.33, 38.35. ESI-MS: m/z calc. for C_10_H_9_FN_2_O_3_S_3_ 342.9657 [M+Na]^+^, found 342.9632 [M+Na]^+^.

### 2.2 Assay for the *in vitro* XO inhibitory potency

XO inhibitory activity was measured by enzyme catalysis reaction *in vitro*, with modifications [28]. The XO solution (100 U/L) and xanthine solution (0.5 mmol/L) were prepared in PBS (10 mmol/L, pH = 7.4) and stored at 4°C. The test compounds were diluted to different concentrations with PBS. The enzyme catalysis reaction was performed in 96-well plates. 100 μL of the test compounds and 50 μL of XO solution were added into 96-well plates and incubated for 5 min at 37°C. Then, the reaction was initiated by the addition of 50 μL xanthine solution and incubated for 30 min at 37°C. The test compounds were replaced with PBS, febuxostat and allopurinol, respectively. All the compounds were tested in triplicate with 5 different concentrations (100, 50, 25, 12.5, 6.25, 3.125 μmol/L). The OD value of each well was measured using microplate reader (America) at 295 nm, and the IC_50_ values were calculated using GraphPad Prism 7.0.

### 2.3 Enzyme inhibitory kinetics

The inhibitory mode of representative compound **6k** was further investigated using enzyme inhibitory kinetic studies. XO solution (55 U/L) was mixed respectively with compound **6k** (0, 5, 10, and 20 μmol/L) and incubated at 37°C for 5 min. Then, xanthine solution (0.25, 0.5, 1.0, and 2.0 mmol/L) were added to initiate the reaction at 37°C for 30 min. The reaction was carried out in a 96-well plate and the OD value were read using a microplate reader at 295 nm. The obtained data were analyzed using Microsoft Excel 2013 and transformed into Lineweaver-Burk plots using GraphPad Prism 7.0. In addition, the value of Ki and Kis could be calculated using GraphPad Prism 7.0.

### 2.4 Molecular modeling

The three-dimensional structure of the protein was downloaded from RCSB Protein Data Bank (www.rcsb.org) Protein Receptor with Ligand Molecule (PDB ID: 3ETR) [29], and the structure of compound 6k was constructed in the MOE module. The protein was processed using Schrödinger’s Protein Preparation Wizard [30], including removed crystal water, added missing hydrogen atoms and repaired missing bond information and peptide fragments. The Ligprep 3.3 module was used to generate stereoisomers of test compound, and the protonation states of ligands at pH 7.0 ± 2.0 were generated with Epik 3.1. Protonation and energy optimization were performed to obtain the 3D configuration using Chem3D Pro 14.0 (PerkinElmer, America). After the grid file was generated, the compound was docked using the Standard Precision mode of Ligand docking in the Glide module, and the optimal configuration was selected for force analysis and plotted with Pymol.

### 2.5 Statistical analysis

Statistical analyses and image processing were performed using Microsoft Excel 2013 (Microsoft Inc., America) and GraphPad Prism 7.0 (GraphPad Software Inc., America). The experiments were repeated three time, and the data in this paper were expressed as mean ±SD. The t-test was used for the comparisons between two groups, p<0.05 was considered a statistically significant difference.

## 3. Results and discussion

### 3.1 Chemical synthesis

**Scheme 1:**
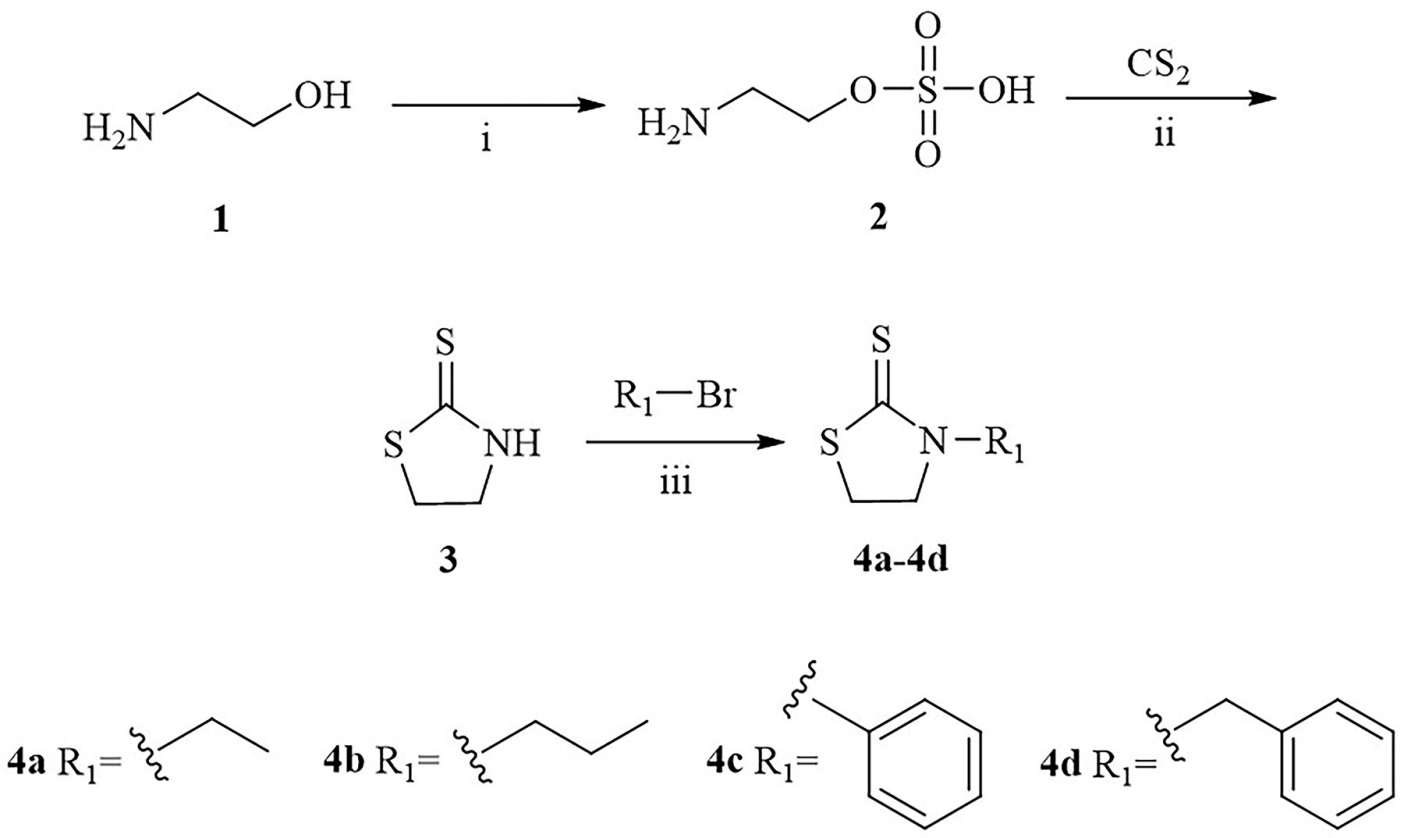
Synthesis of thiazolidine-2-thione derivatives 4a-4d. i: H_2_SO_4_, 0°C; ii: KOH, EtOH, 40°C; iii: NaOH, EtOH, 50°C (**4a, 4b**); NaOH, EtOH, CuI, 80°C (**4c, 4d**).

**Scheme 2:**
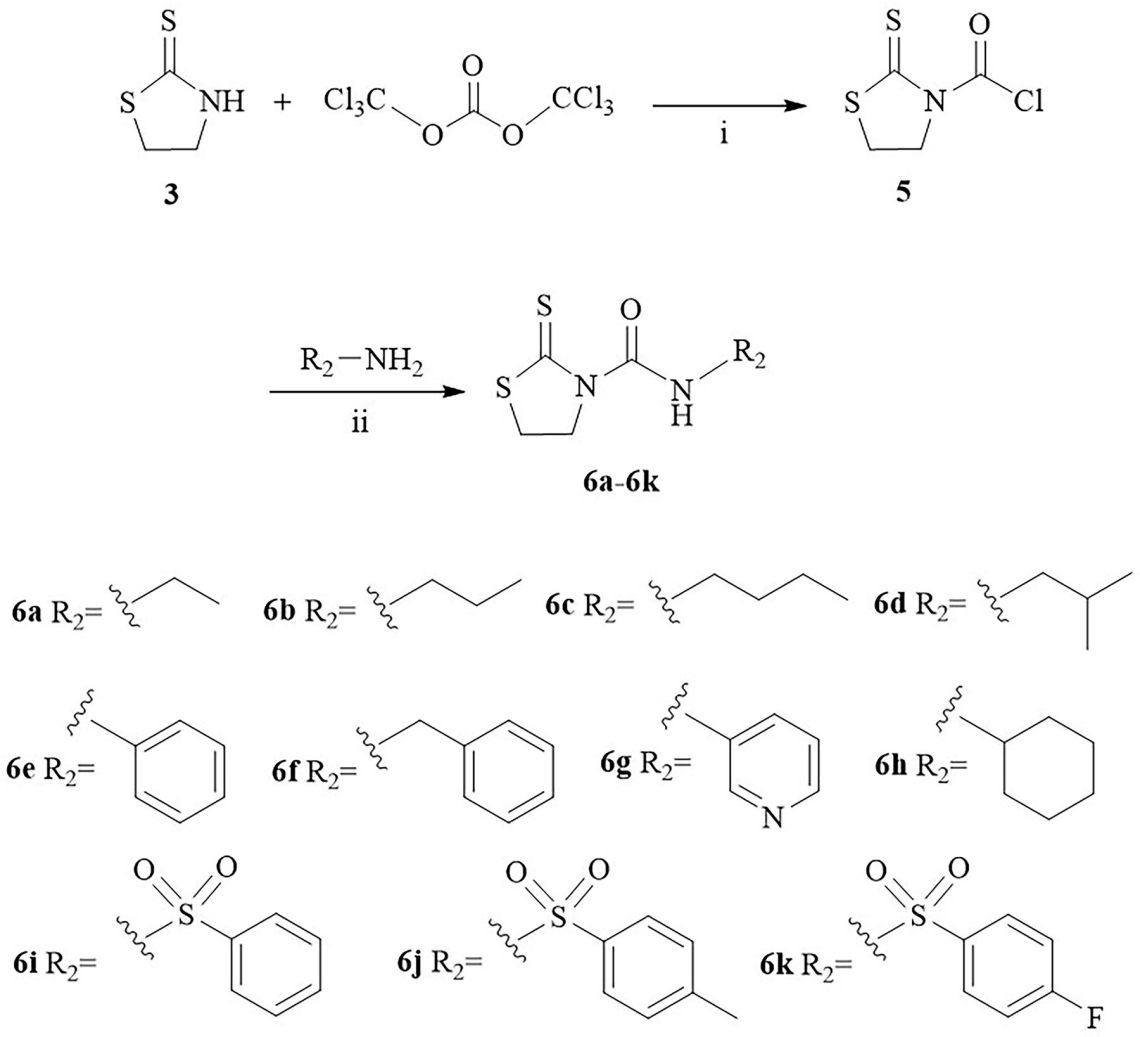
Synthesis of thiazolidine-2-thione derivatives 6a-6k. i. TEA, CH_2_Cl_2_, 0°C; ii. TEA, THF, R.T.

A series of thiazolidine-2-thione derivatives were prepared in this paper, and the synthetic routes were shown in Schemes 1, 2. Thiazolidine-2-thione (**3**) was prepared according to previous method [31] with a slight modification using aminoethanol as the starting material. Compounds **4a-4d** were synthesized from thiazolidine-2-thione and ethyl bromide/bromopropane/bromobenzene/benzyl bromide by utilizing NaOH or NaOH+CuI as catalyst. Thiazolidine-2-thione was reacted with triphosgene in anhydrous CH_2_Cl_2_, and a variety of amines were added to obtain the compounds **6a-6k** in the presence of triethylamine. The structures of the obtained derivatives were confirmed (S1 Fig in S1 File) by nuclear magnetic resonance (^1^H NMR and ^13^C NMR) and high-resolution electrospray ionization mass spectrometry (HR-ESI-MS).

### 3.2 *In vitro* XO inhibitory activity

The XO inhibitory activity of all derivatives was determined by *in vitro* enzyme catalysis reaction with allopurinol and febuxostat as positive control, and the results were shown in Table 1. Compared with thiazolidine-2-thione (IC_50_ = 72.15 μmol/L), most of the derivatives shown significant XO inhibitory activity with IC_50_ values between 3.56 μmol/L and 58.17 μmol/L, whereas compounds **4d** demonstrated weak inhibitory activity with IC_50_ values exceeding of 100 μmol/L. Among them, compounds (**6i**- **6k**) containing phenyl-sulfonamide in the structure exhibited the more potent XO inhibitors, revealing IC_50_ values of 5.19, 9.76, and 3.56 μmol/L, respectively. In particular, compound **6k** exhibited an XO inhibitory activity similar to that of febuxostat (IC_50_ = 3.34 μmol/L), which was approximately 2.5-fold more potent than that of allopurinol (IC_50_ = 7.86 μmol/L). Structure-activity relationship indicated that introduction of the amide group significantly enhanced the inhibitory activity of compound (**4a** vs **6a**; **4b** vs **6b**; **4c** vs **6e**; **4d** vs **6f**;), which was consistent with the results of previous studies [32]. In addition, introduction of benzene sulfonamide substituted by electron-donating group could decrease the inhibitory activity (**6i** vs **6j**), whereas the substituted by electron-withdrawing group could obviously enhance the inhibitory activity (**6i** vs **6k)**, which meant that the electron-withdrawing group linked at the benzene sulfonyl was more preferable for the XO inhibitory activity [33].

**Table 1:** *In vitro* XO inhibitory potency of thiazolidine-2-thione derivatives.

Compound	IC_50_ (μmol/L)	Compound	IC_50_ (μmol/L)
**3**	72.15 ± 1.66	**6e**	10.20 ± 1.37
**4a**	43.53 ± 2.18	**6f**	12.80 ± 1.60
**4b**	58.17 ± 6.71	**6g**	14.52 ± 2.90
**4c**	51.60 ± 1.64	**6h**	9.87 ± 1.19
**4d**	>100	**6i**	5.19 ± 0.95
**6a**	22.90 ± 2.24	**6j**	9.76 ± 1.29
**6b**	27.80 ± 0.63	**6k**	3.56 ± 0.61
**6c**	30.54 ± 2.25	Allopurinol	7.86 ± 0.91
**6d**	19.04 ±1.44	Febuxostat	3.34 ± 0.53

IC_50_ values were expressed as the mean±SD.

### 3.3 Enzyme inhibitory kinetics analysis of compound 6k

**Fig 1:**
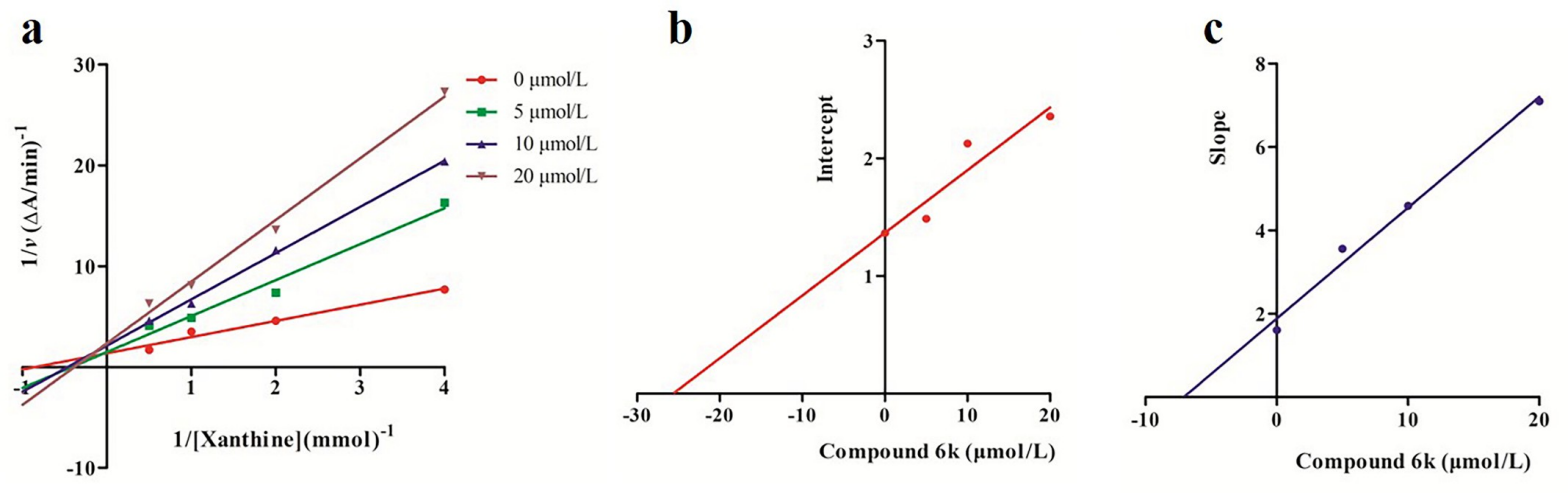
Kinetic analysis of compound 6k inhibited of XO activity. (a) Lineweaver-Burk plots analysis, c(XO) = 55 U/L, c(compound **6k**) = 0, 5, 10, and 20 μmol/L, c(xanthine) = 0.25, 0.5, 1.0, and 2.0 mmol/L. (b/c) *Ki* and *Kis* of compound **6k**, *Ki* and *Kis* were obtained from secondary plots of the slopes of the Lineweaver-Burk plots and the apparent 1/Vmax versus the inhibitor concentrations, respectively.

To research the inhibitory mode of compound **6k**, enzyme inhibitory kinetic studies were performed and the inhibitory mode were analyzed using Lineweaver-Burk plots. As shown in Fig 1, the changed of compound **6k** concentration resulted in the changes of slope and Y-intercept, suggesting that Km and Vmax were changed with the change the concentration of compound **6k**. The curve intersected in the first quadrant, showing that Km increased and Vmax decreased with the increasing of compound **6k** concentration. The results confirmed that the mode of XO inhibition by compound **6k** belonged to mixed competitive inhibition, which was different from allopurinol with a competitive inhibition [34]. At the same time, the values of *Ki* and *Kis* were calculated based on the Lineweaver-Burk plot, which *Ki* was competitive inhibition constant for binding with free enzyme and *Kis* was noncompetitive inhibition constant for binding with enzyme-substrate complex. The results showed that the *Ki* and *Kis* values of compound **6k** were 7.08 μmol/L and 25.67 μmol/L, respectively, which suggested that compound **6k** preferentially bound to the free XO rather than to the XO-xanthine complex.

### 3.4 Molecular modeling study of compound 6k

**Fig 2:**
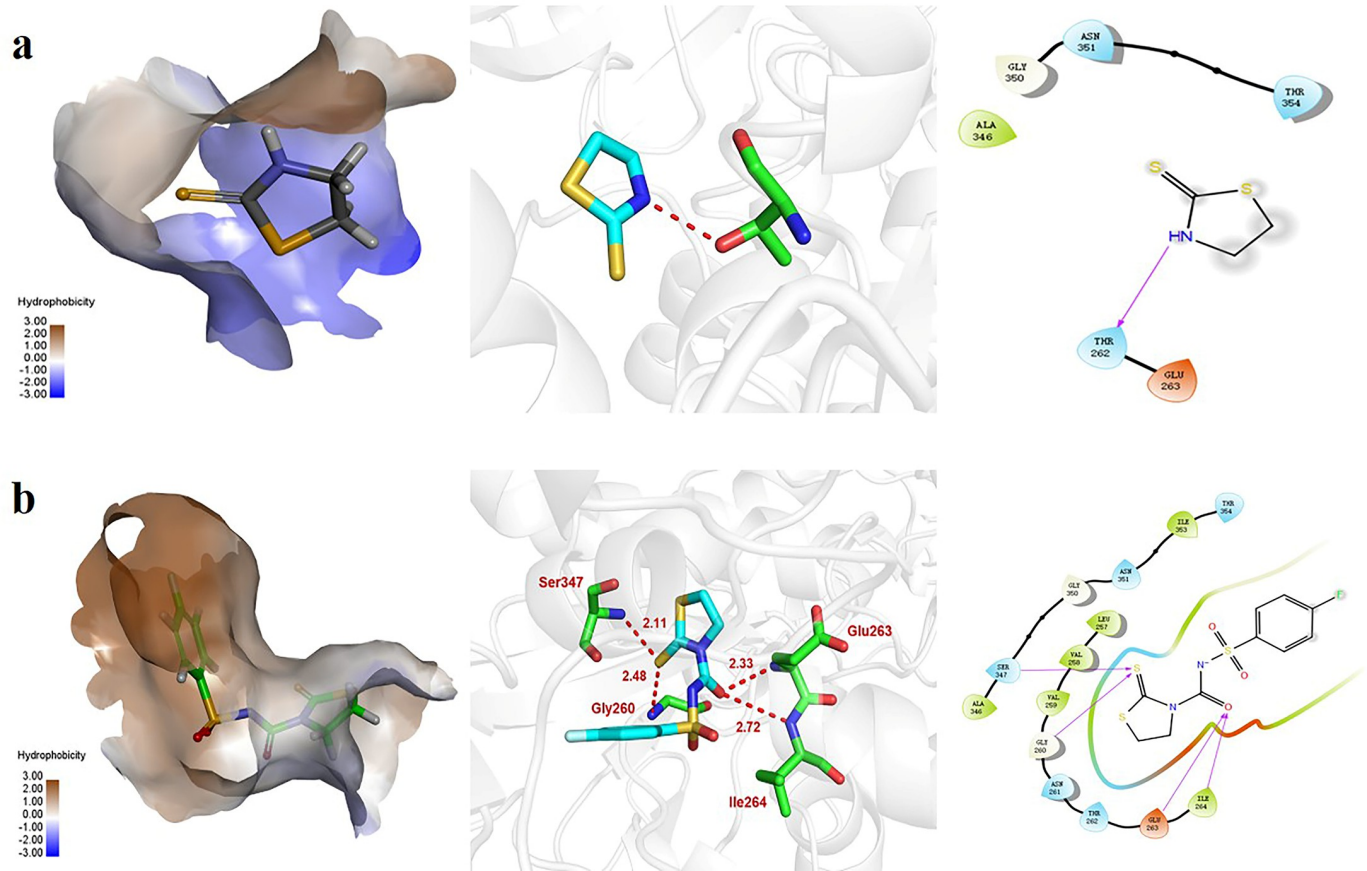
Molecular docking of thiazolidine-2-thione and compound 6k within the binding pocket of XDH. (a) The binding modes of thiazolidine-2-thione with XDH (PDB: 3ETR), and the hydrogen bonds of thiazolidine-2-thione with the key amino acid residues in XDH; (b) The binding modes of compounds **6k** with XDH, and hydrogen bonds of compound **6k** with the key amino acid residues in XDH.

To further research the potential binding mode between enzyme and drug molecule, molecular docking study of thiazolidine-2-thione and compound **6k** were performed in this paper. Since there is no complete crystal structure and the molybdenum-pterin centers of both xanthine dehydrogenase (XDH) and XO are identical in terms of binding modes and substrate catalysis [35], so XDH crystal structure (PDB: 3ETR) was used in molecular docking research. The affinity of thiazolidine-2-thione and enzyme was -6.68 kcal/mol, and the affinity of compound **6k** and enzyme was -10.3 kcal/mol, which suggested that compound **6k** presented a more compact binding mode in the enzyme active pocket. As shown in Fig 2A, thiazolidine-2-thione formed a hydrogen bond with the amino acid residue Thr262 of the enzyme with a distance of 1.97 Å. Fig 2B shown that compound **6k** was accommodated in the active site through hydrogen bonds with primary amino acids, including Gly260, Glu263, Ile264 and Ser347, and the interaction distances were 2.48 Å, 2.33 Å, 2.72 Å and 2.11 Å, respectively. Among them, the 4-fluorophenyl-sulfonyl moiety interacted with the amino acid residue of the enzyme active pocket *via* 2 hydrogen bonds, in which the carbonyl group acted as a hydrogen bond acceptor interacting with the amino group of Gly260 and Ile264, respectively. At the same time, the thiazolidinethione moiety formed two hydrogen bonds with the amino acid residue of Glu263 and Ser347 in the enzyme hydrophobic cavity, which was similar to 2-(indol-5-yl) thiazole derivatives [36]. The molecular docking results explained why compound **6k** could produce more potent XO inhibitory activity than thiazolidine-2-thione.

## 4. Conclusion

In summary, a series of novel thiazolidine-2-thione derivatives were designed and synthesized as XO inhibitors, and the inhibitory activity was evaluated in this study. The results of *in vitro* enzyme catalysis shown that compound **6k** was the most effective XO inhibitor with an IC_50_ value of 3.75 μmol/L. Structure-activity relationship analysis revealed that the phenyl-sulfonamide group was indispensable for compound **6k** to produce XO inhibitory activity. The enzyme inhibition kinetics analyses confirmed that compound **6k** exerted a mixed-type XO inhibitor. In addition, molecular docking studies shown that compound **6k** could bind tightly to the active pocket of the enzyme through hydrogen bonds, and the affinity of compound **6k** and enzyme was -10.3 kcal/mol. Accordingly, the results described above suggested that compound **6k** could be a valuable lead compound for the treatment of hyperuricemia.

## Supporting information

S1 File (DOCX)
