# Supplementary material for: Synthesis and biological evaluation of thiazolidine-2-thione derivatives as novel xanthine oxidase inhibitors
Source: PLoS One. 2022 May 18;17(5):e0268531. doi: 10.1371/journal.pone.0268531 (PMC9116648; doi:10.1371/journal.pone.0268531)
Supplement: S1 File — (DOCX) [file pone.0268531.s001.docx]

**Supporting Information**

**Synthesis and biological evaluation of thiazolidine-2-thione derivatives as novel**

**xanthine oxidase inhibitors**

Mu-xuan Wang^a,1^, Hong-wei Qin^c,1^, Chao Liu^b*^, Shen-ming Lv^a^, Jia-shu Chen^b^,

Chun-gu Wang^a^, Ying-ying Chen^b^, Jia-wei Wang^a^, Jin-yue Sun^b*^, and Zhi-xin Liao^a*^

^a^ Department of Pharmaceutical Engineering, School of Chemistry and Chemical Engineering and Jiangsu Province Hi-Tech Key Laboratory for Biomedical Research, Southeast University, Nanjing, Jiangsu 211189, P.R. China.

^b^ Key Laboratory of Novel Food Resources Processing, Ministry of Agriculture and Rural Affairs/Key Laboratory of Agro-Products Processing Technology of Shandong Province/Institute of Agro-Food Science and Technology, Shandong Academy of Agricultural Sciences, Jinan, Shandong 250100, P.R. China.

^c^ School of Life Sciences and Bioengineering, Jining University, Qufu, Shandong 273155, P.R. China.

*Co-author E-mail: zxliao@seu.edu.cn (Zhixin Liao), liuchao555@126.com (Chao Liu), moon_s731@hotmail.com (Jin-yue Sun).

^1^ These authors contributed equally to this work.

**S1 Fig. ^1^H NMR, ^13^C NMR and HR-ESI-MS of all compounds**

Compound **2**


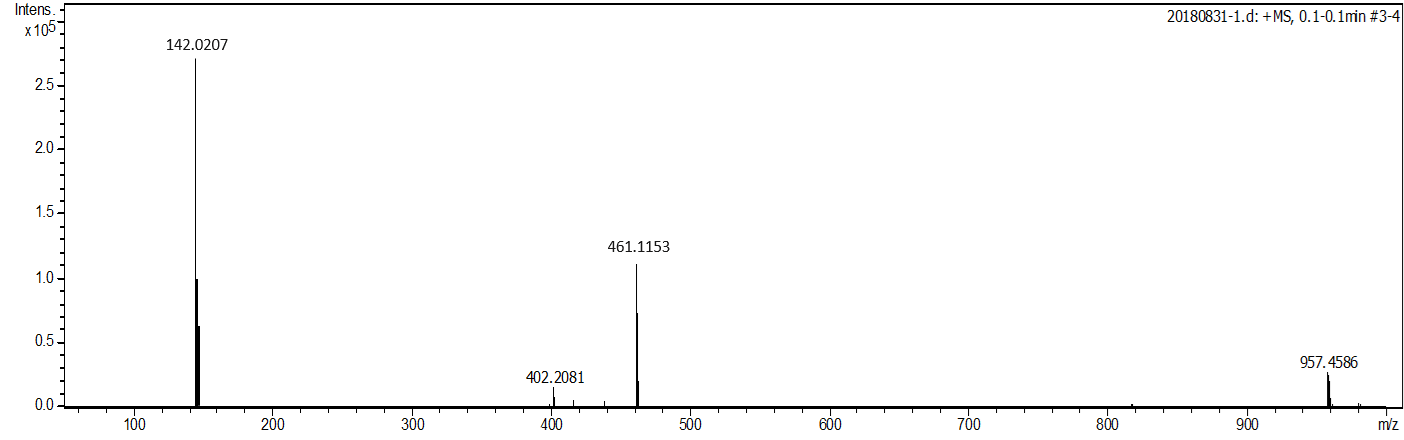


Compound **3**


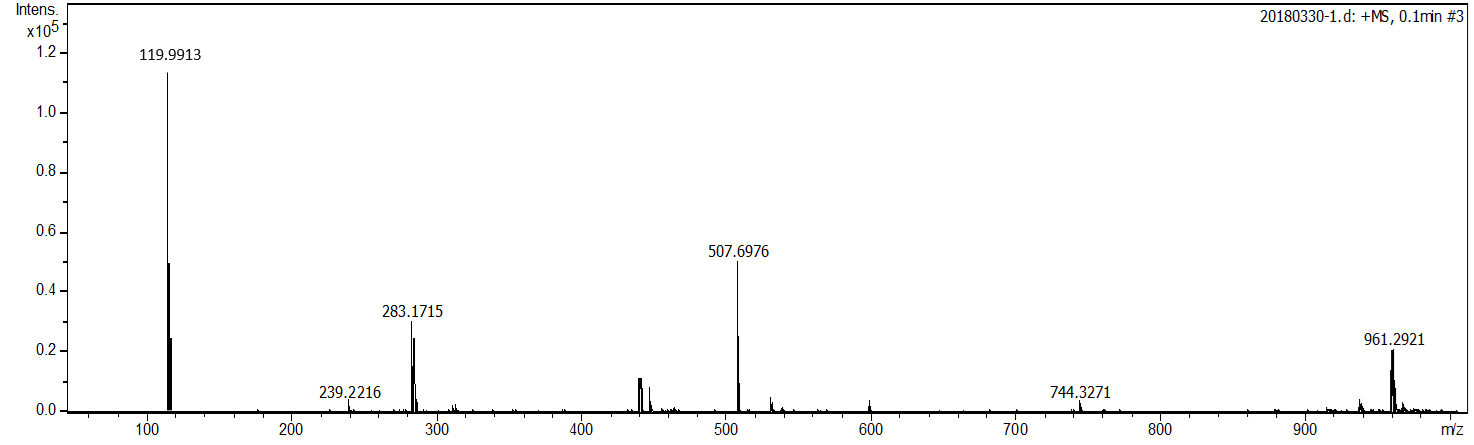


Compound **4a**


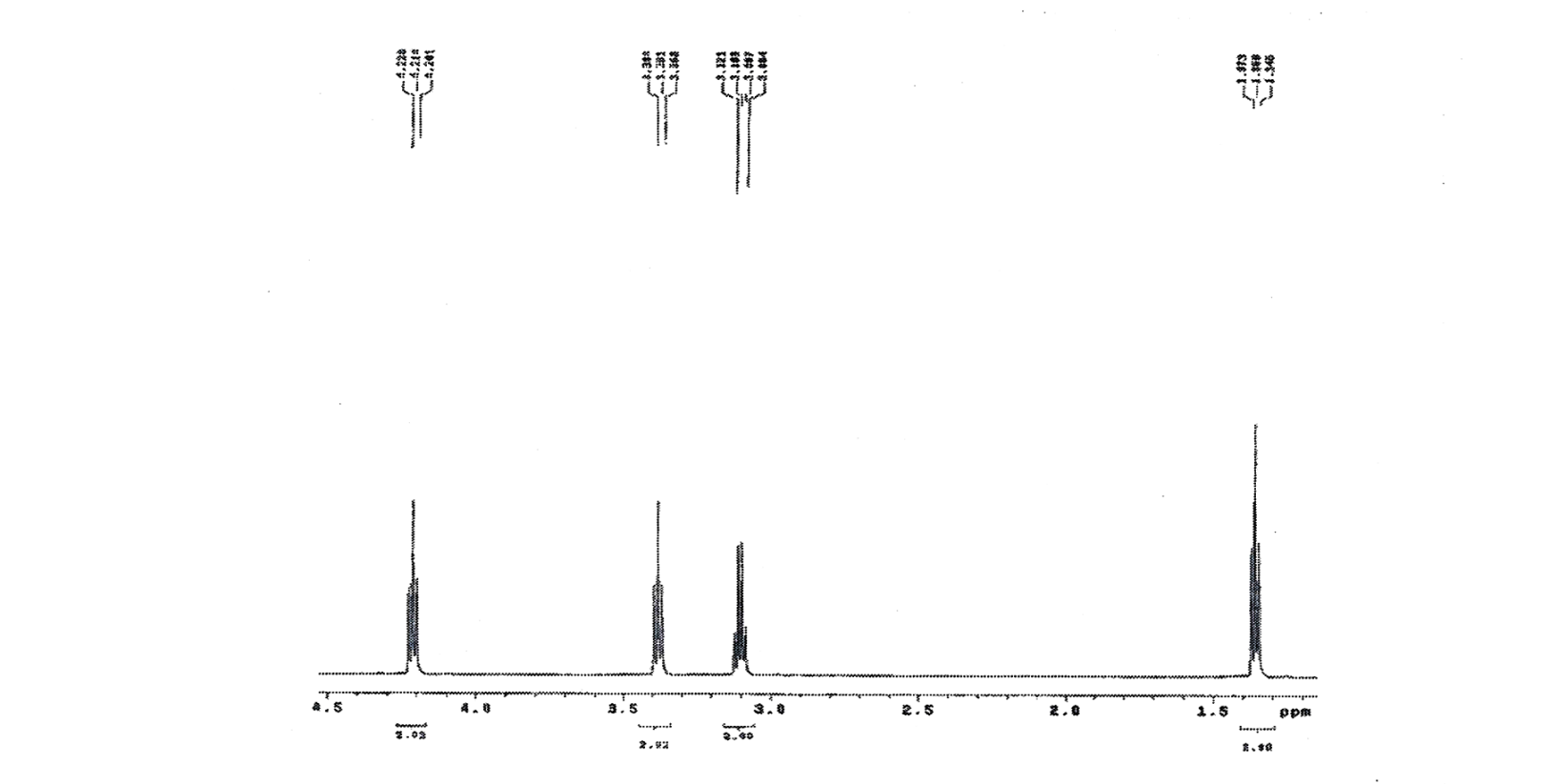


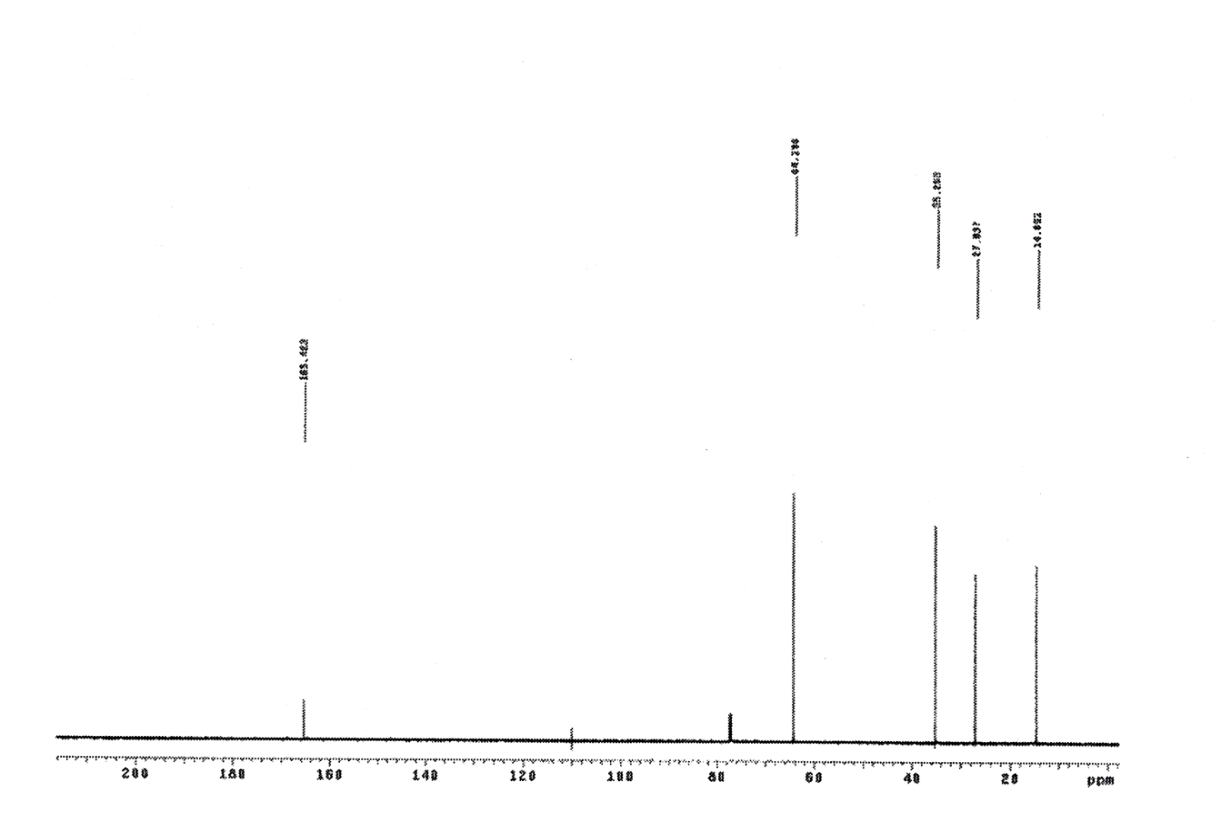


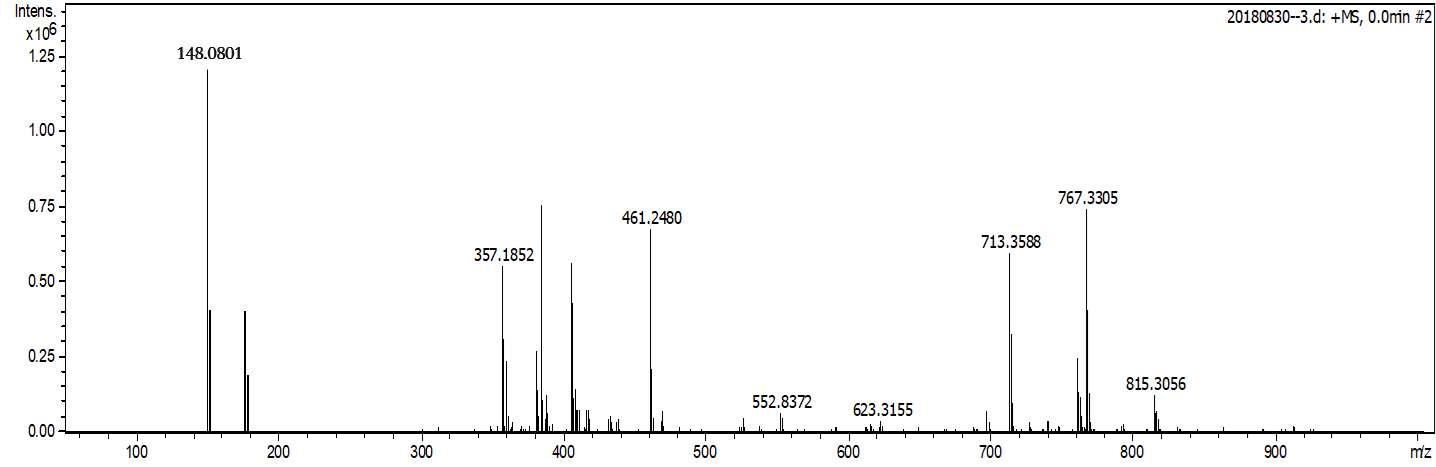


Compound **4b**

**
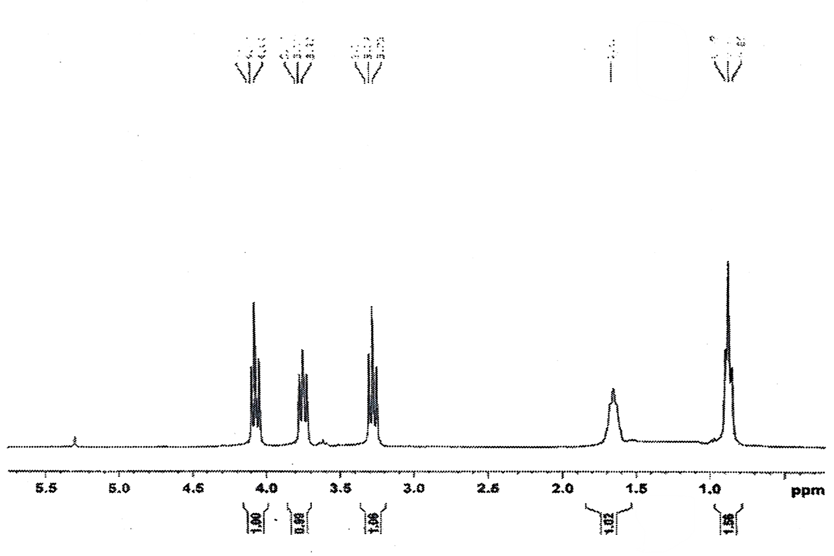
**


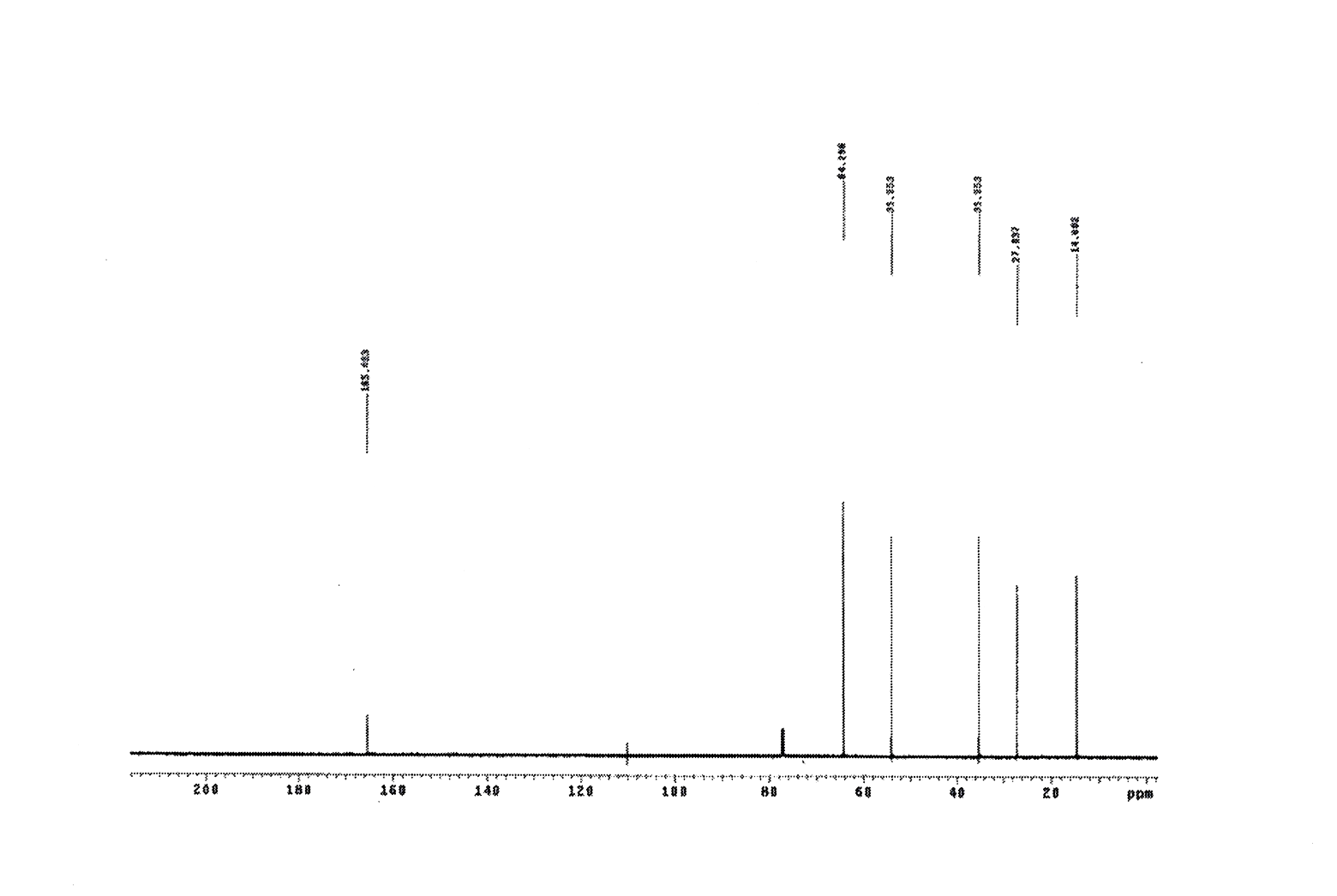


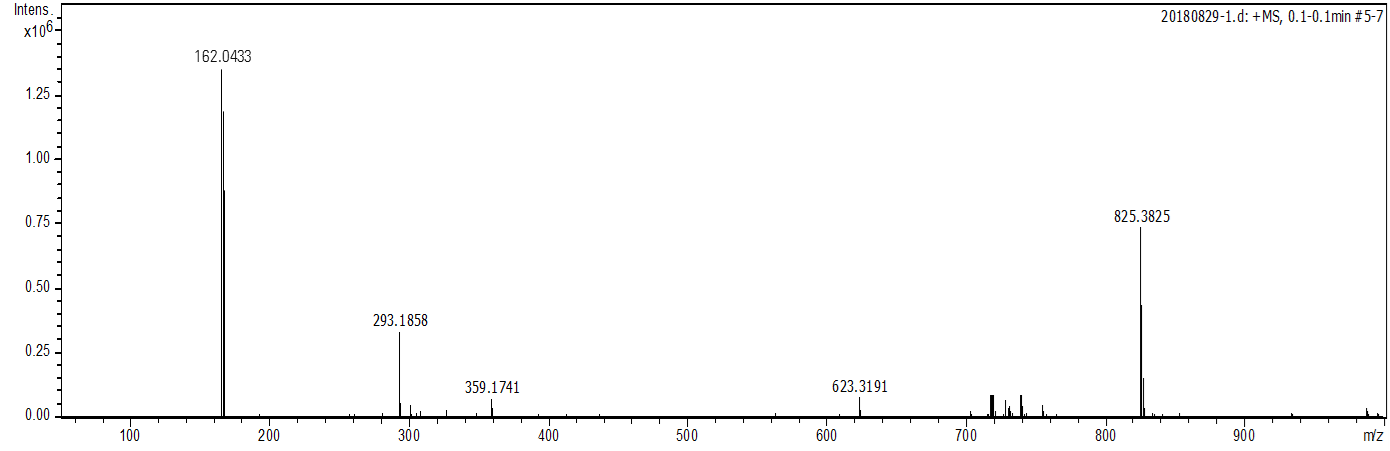


Compound **4c**


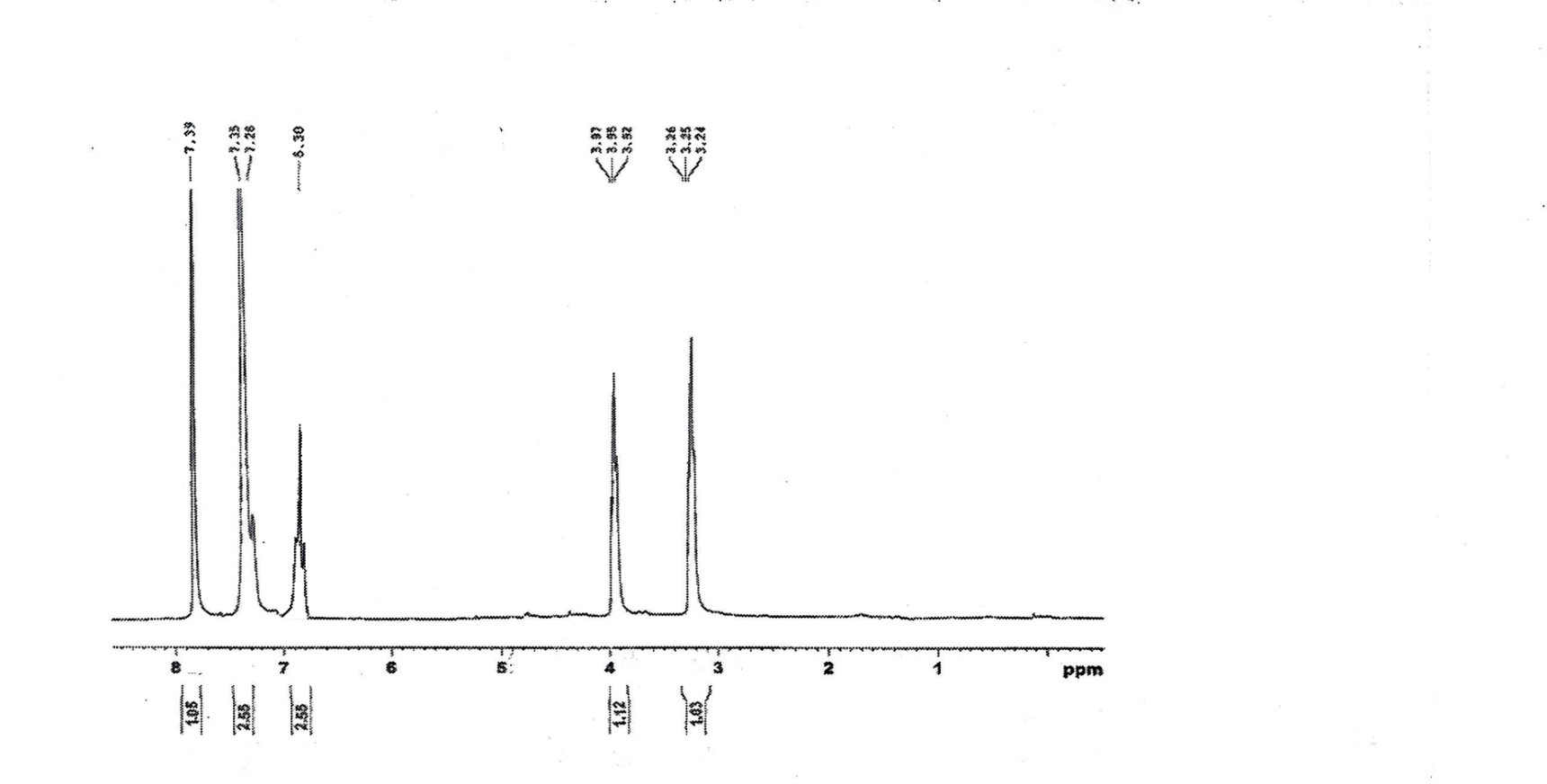


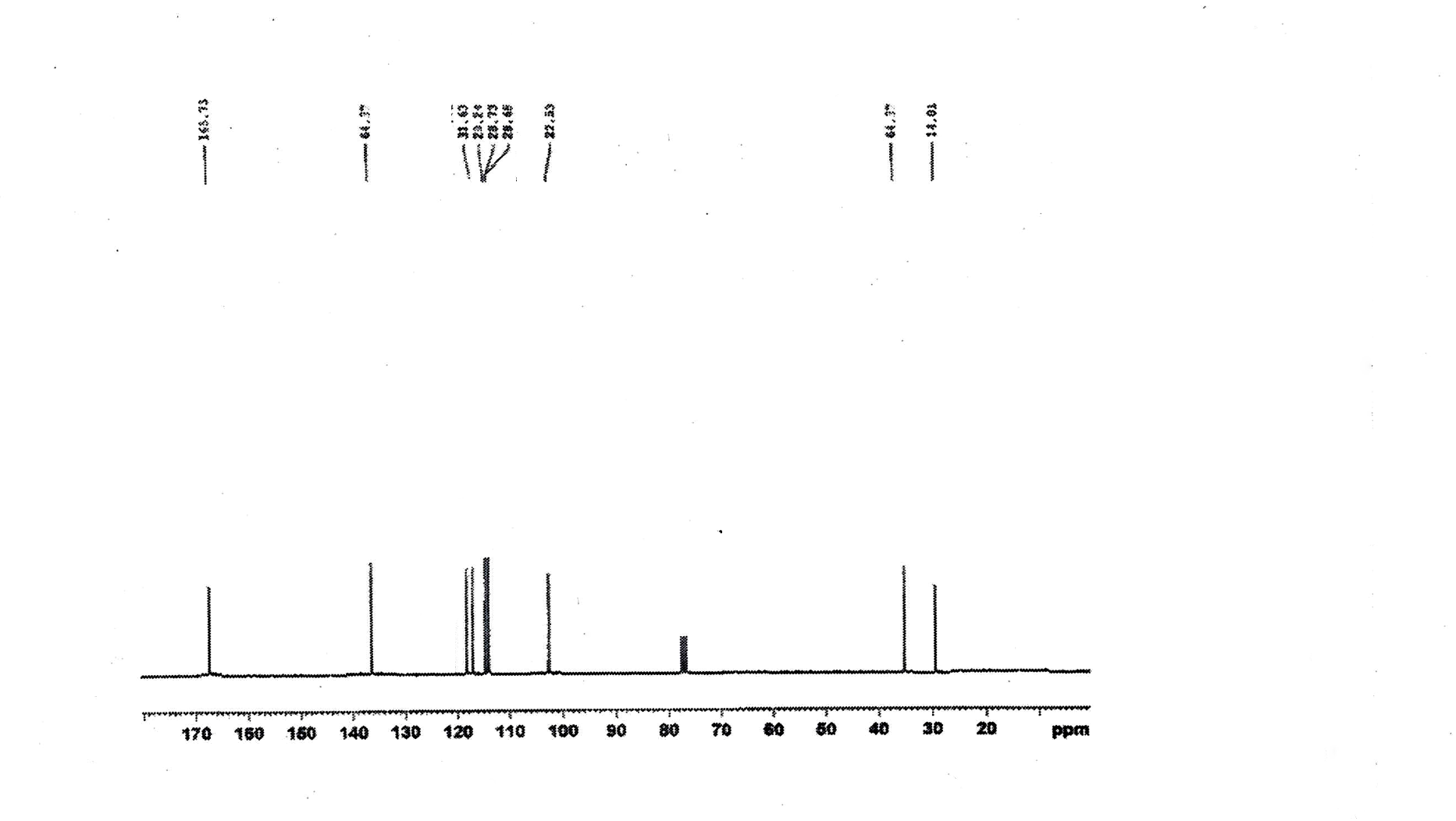


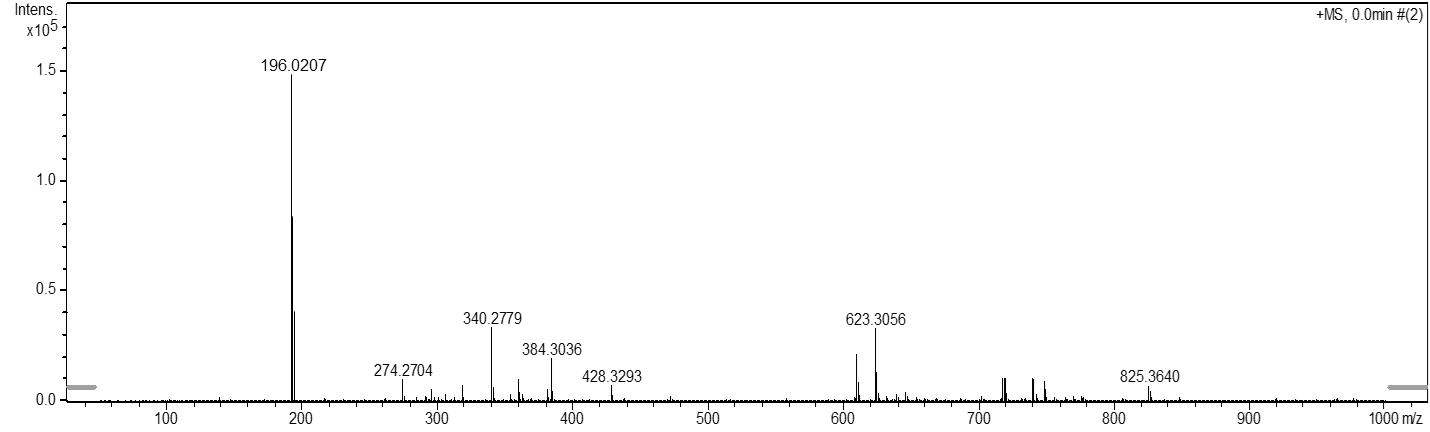


Compound **4d**


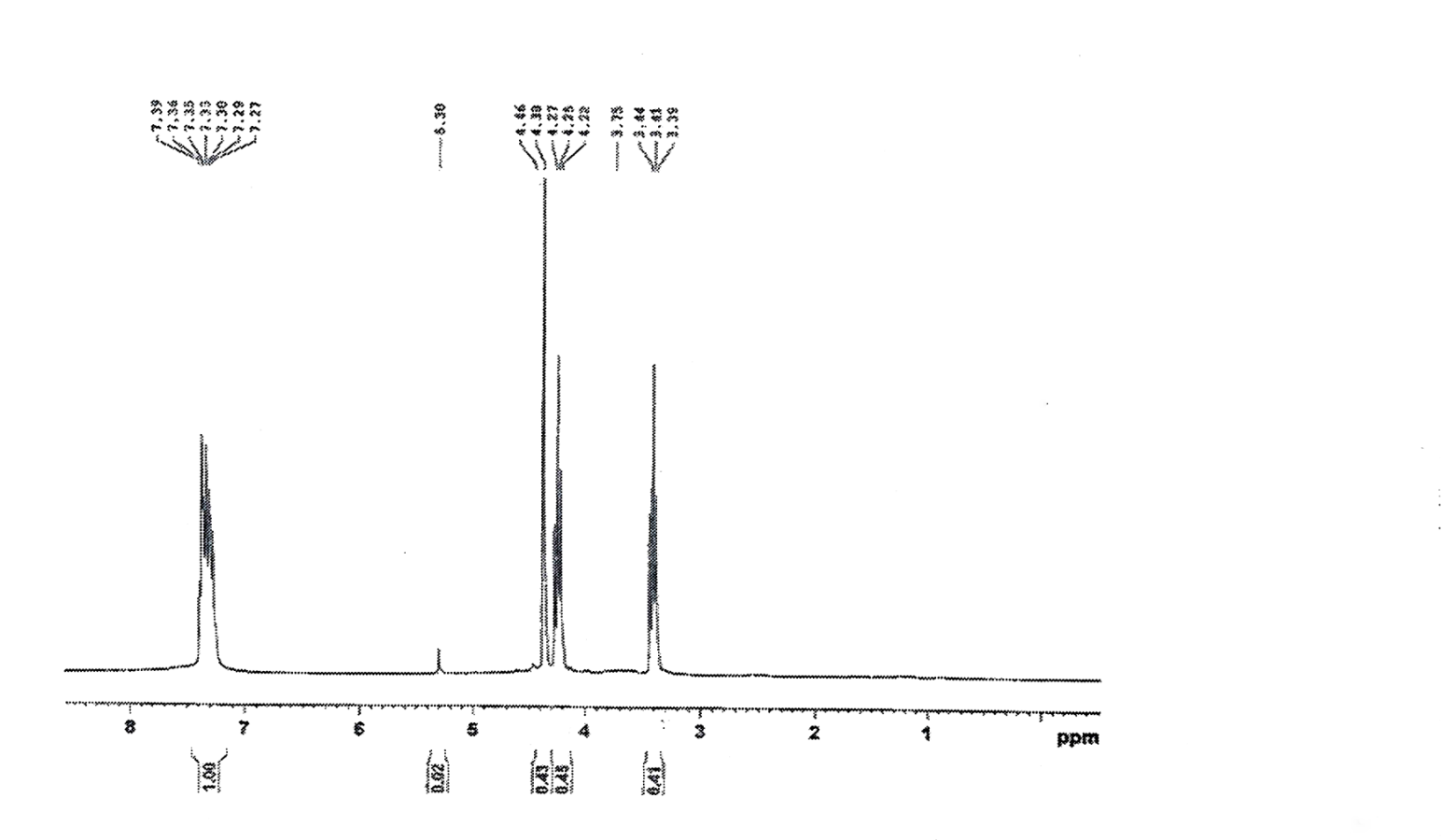


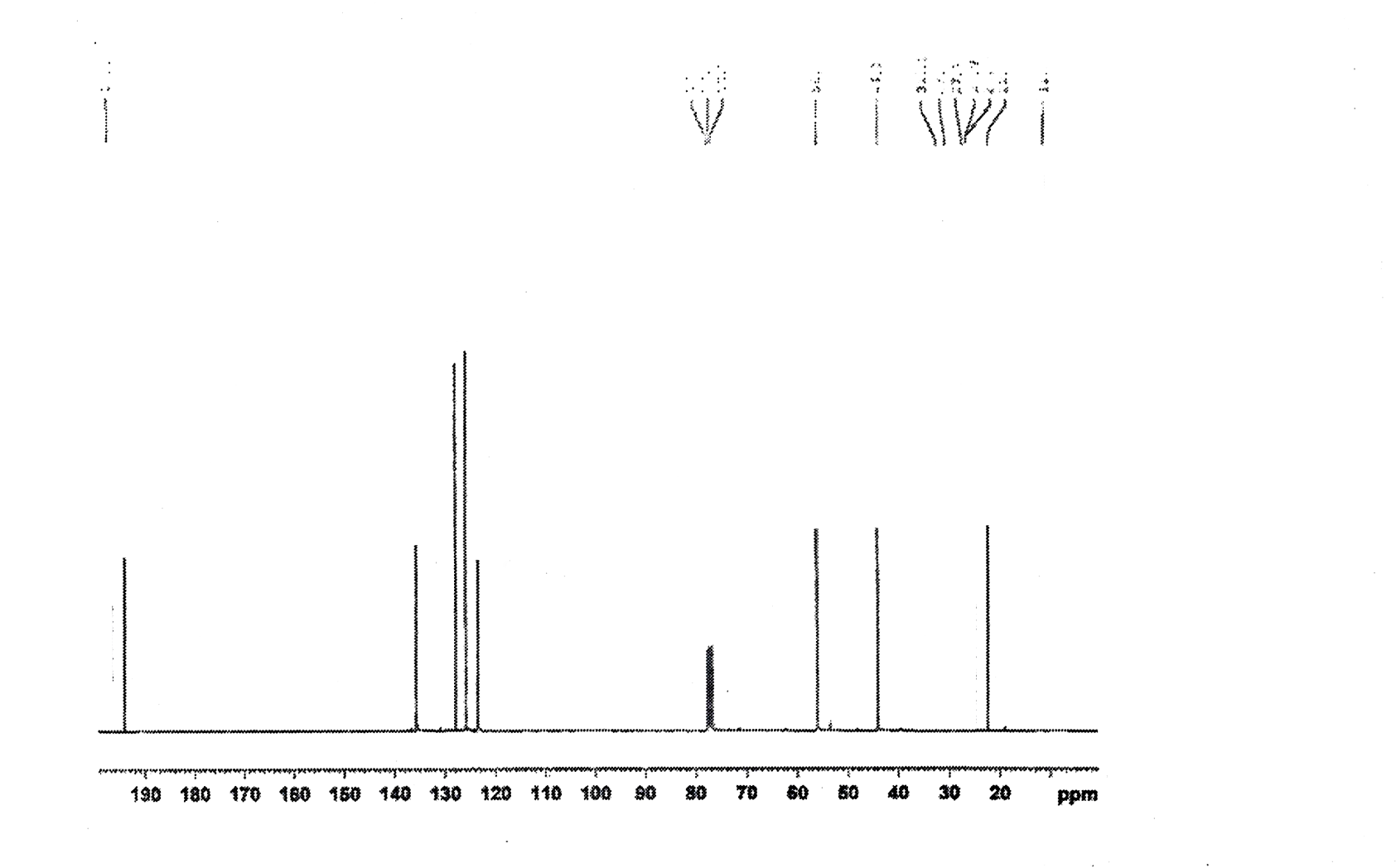


**
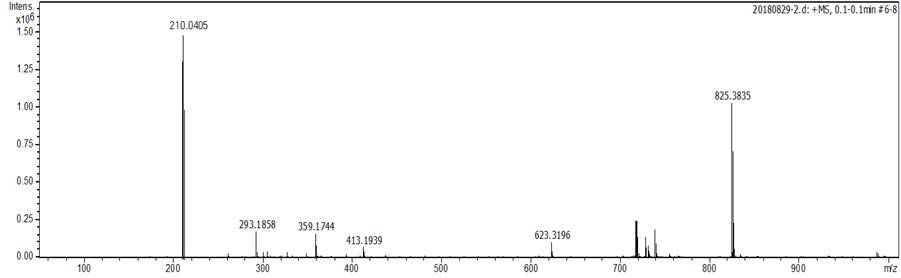
**

Compound **6a**


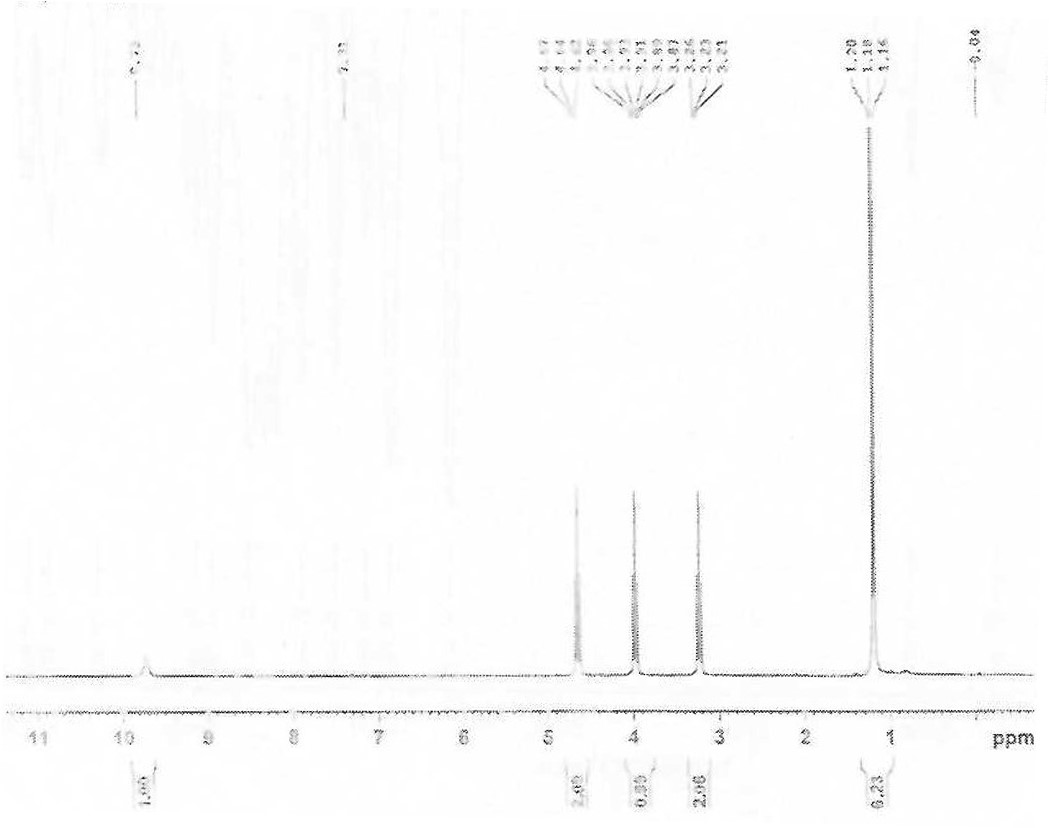


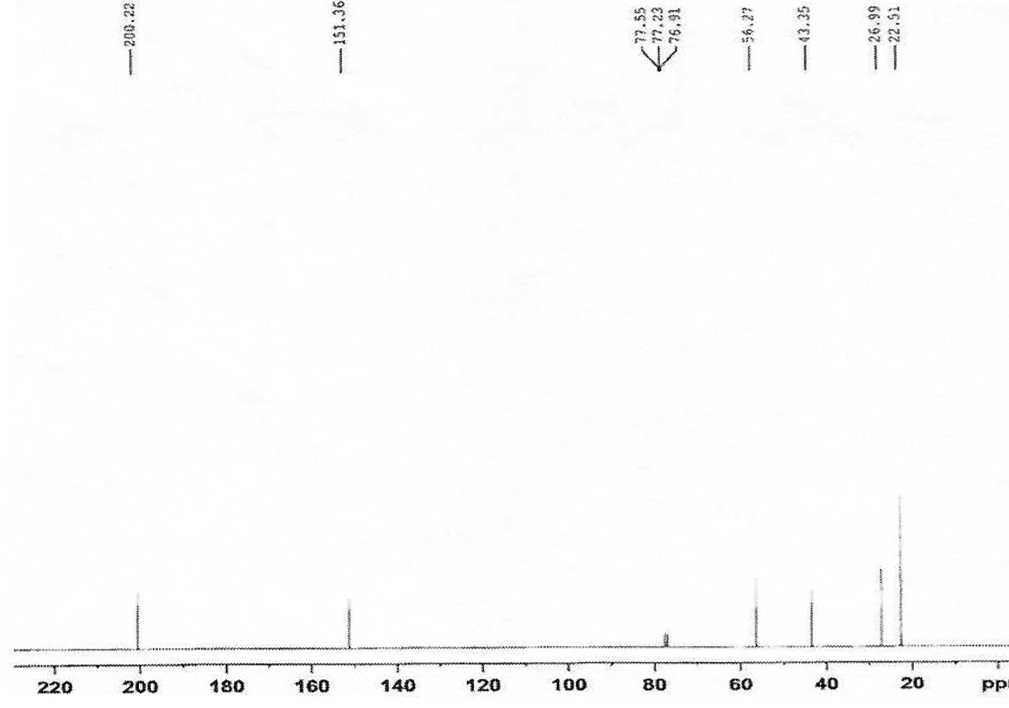


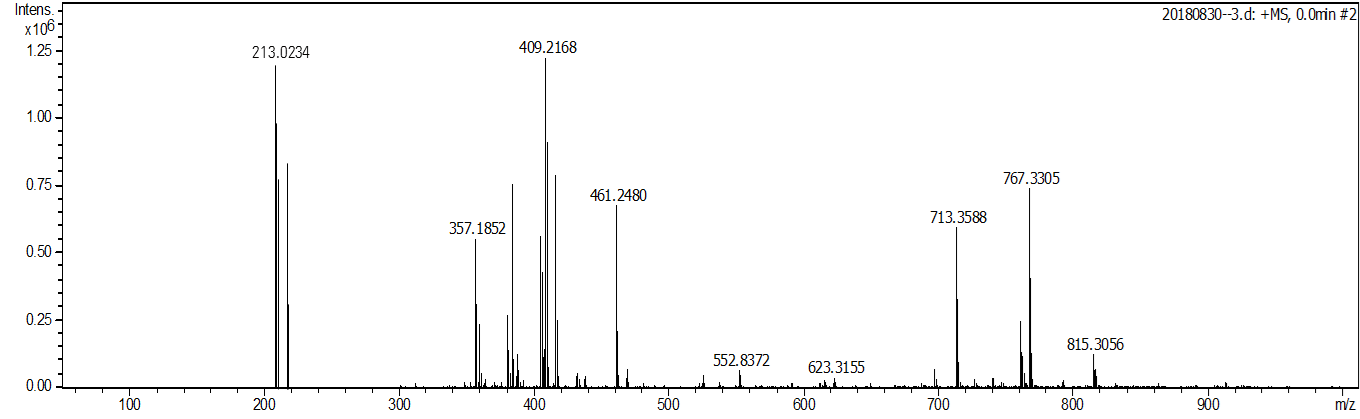


Compound **6b**


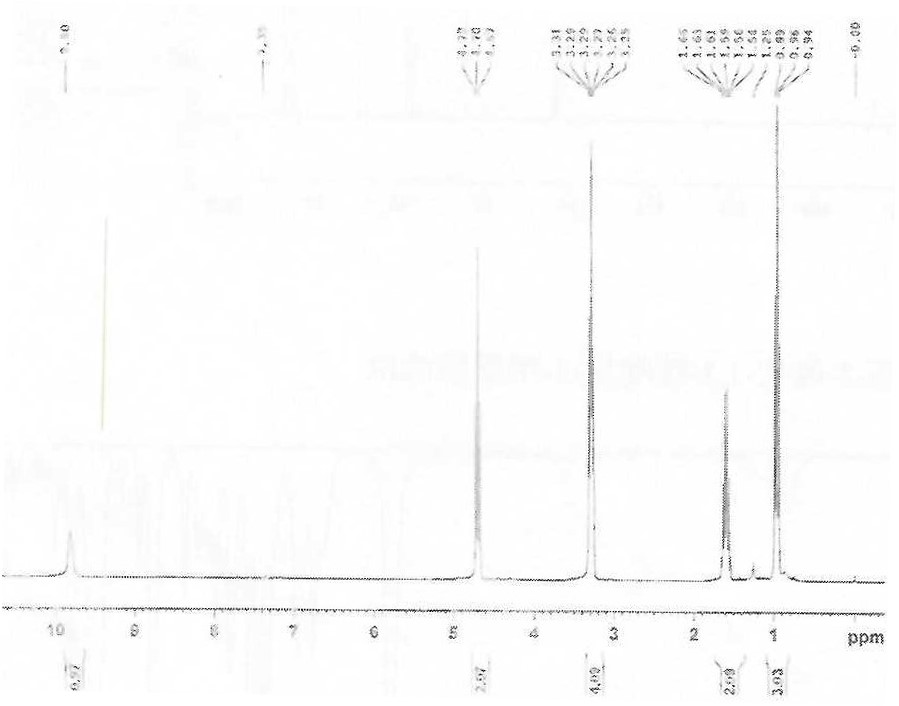


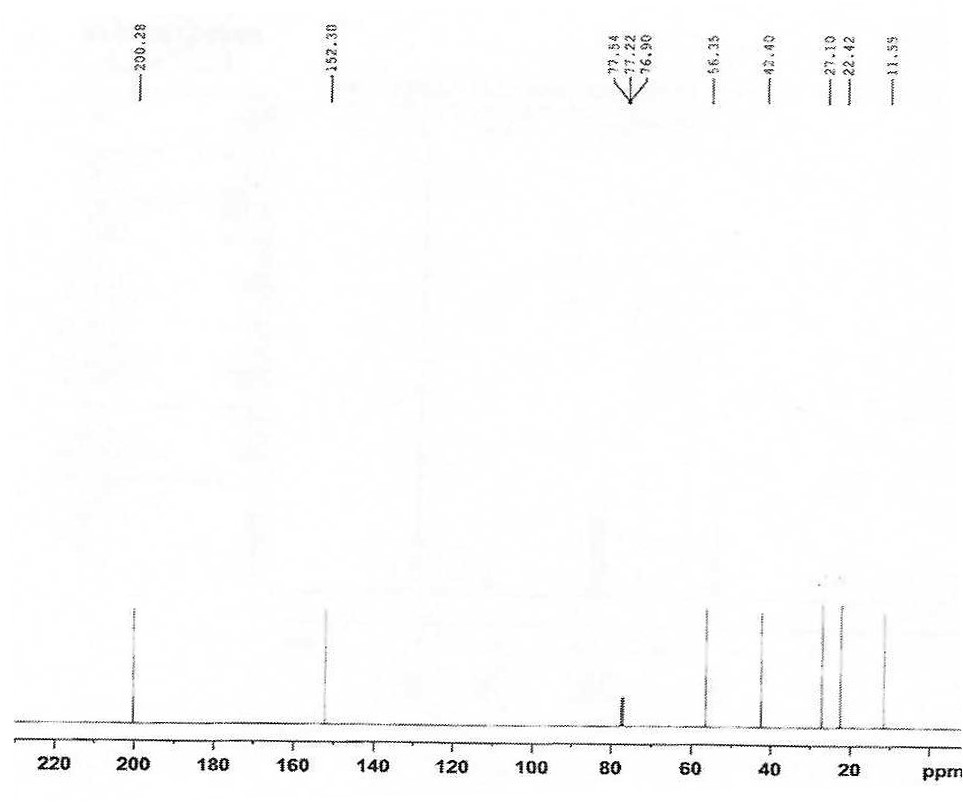


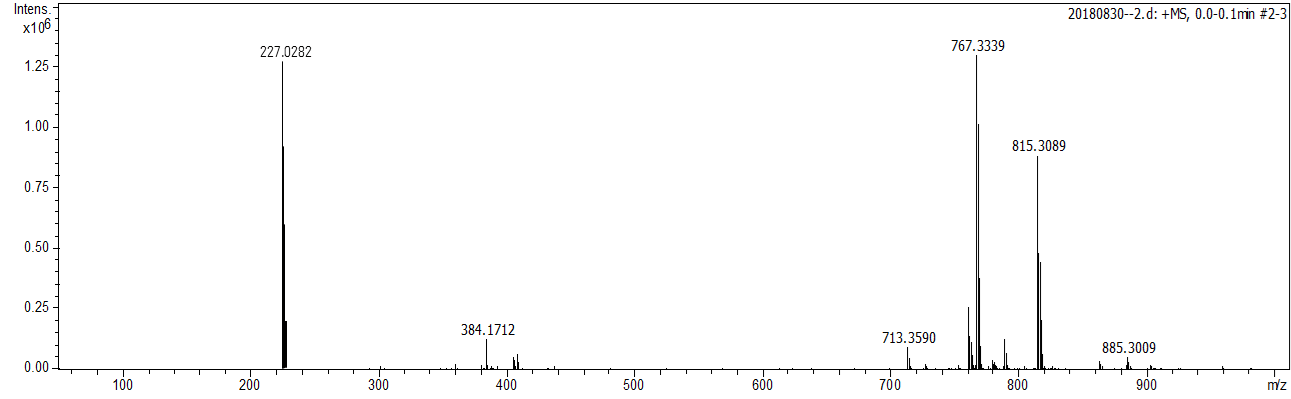


Compound **6c**


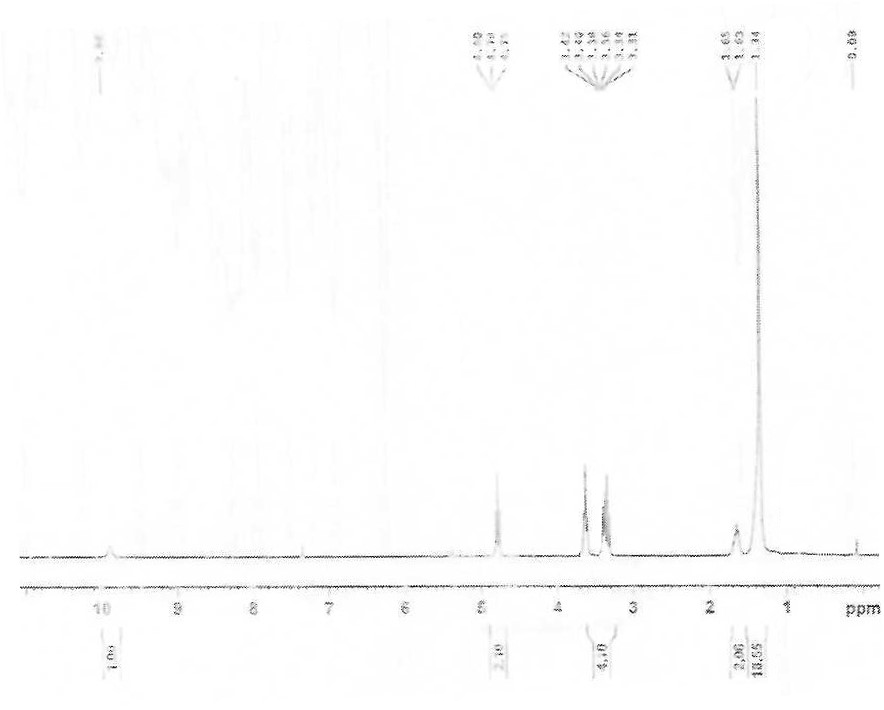


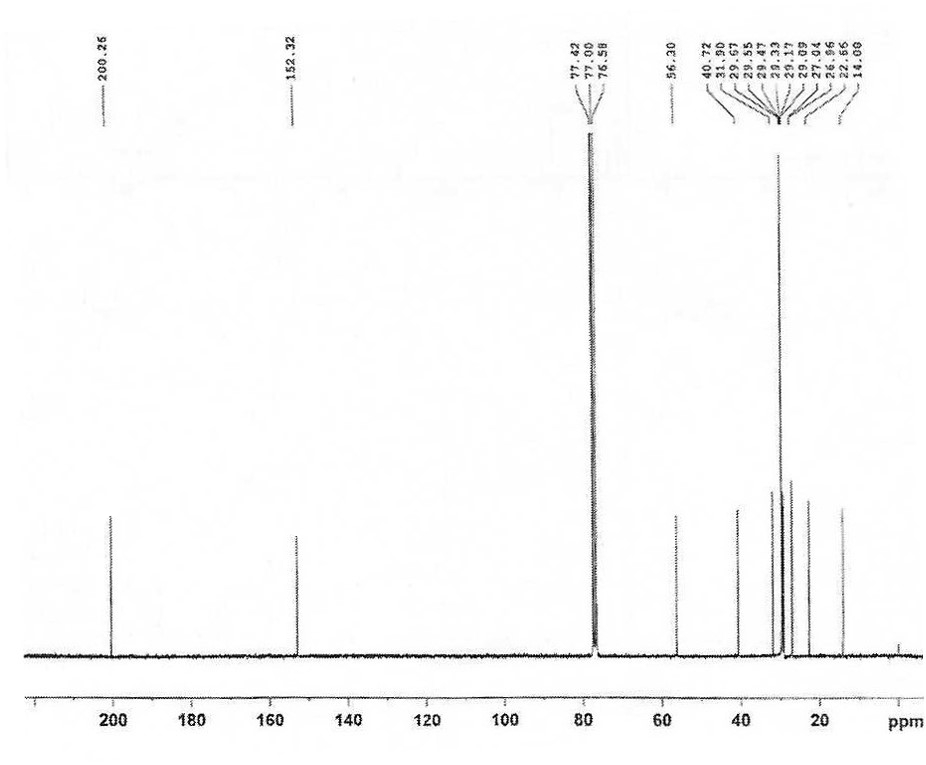


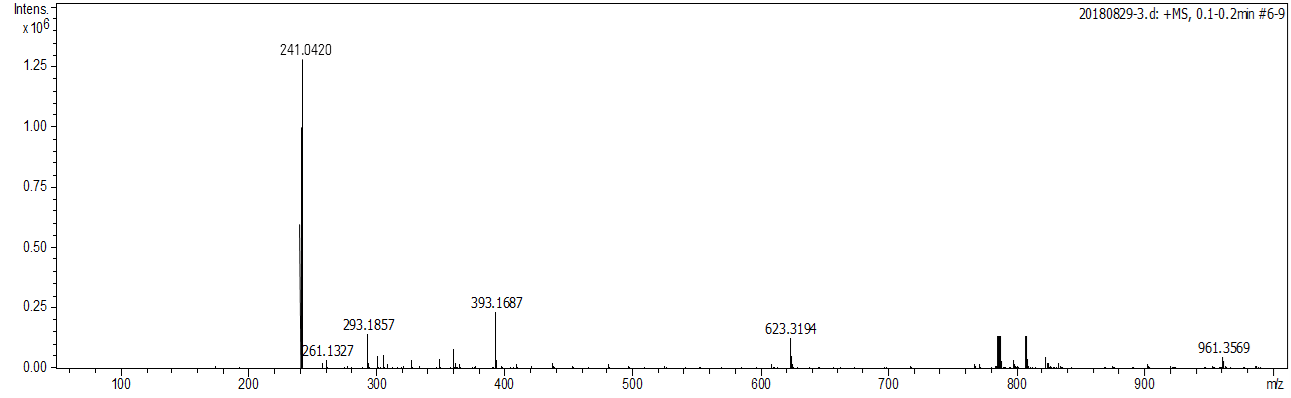


Compound **6d**


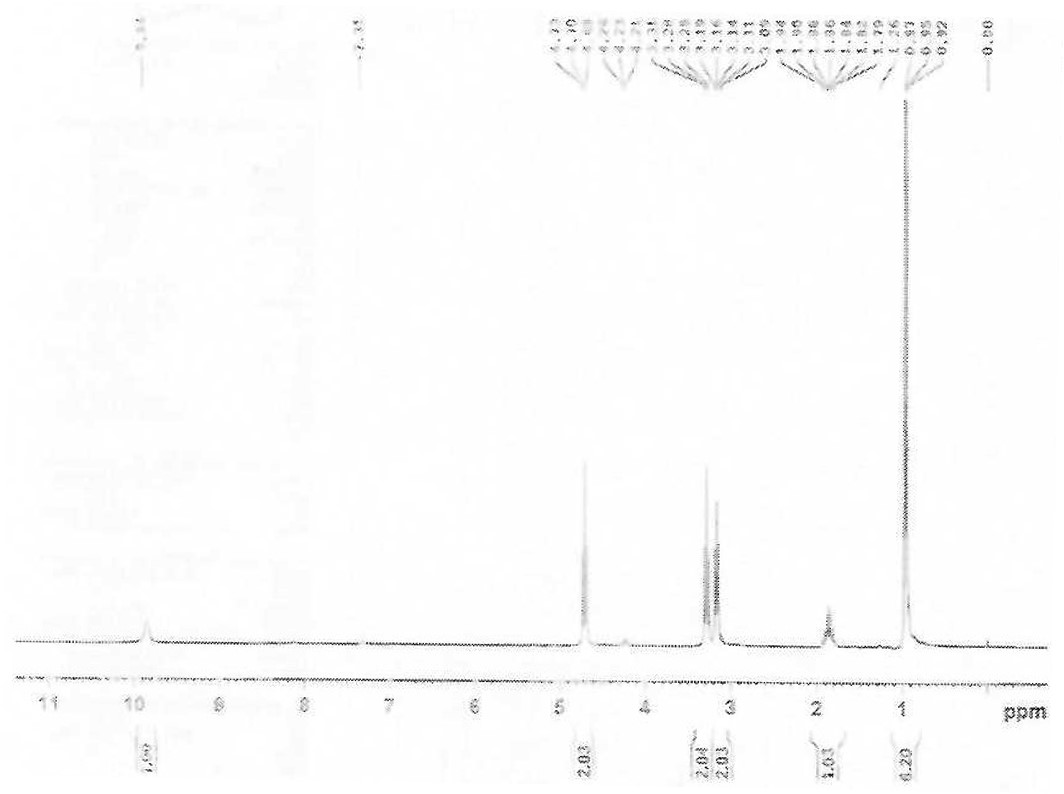


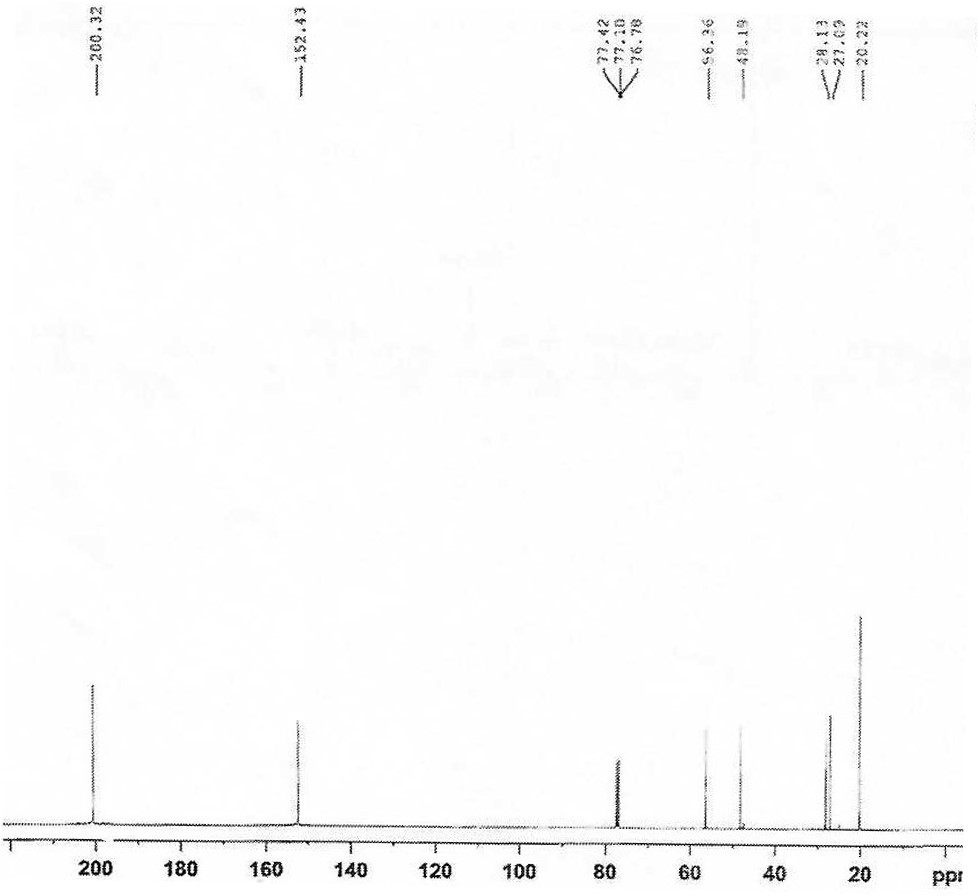


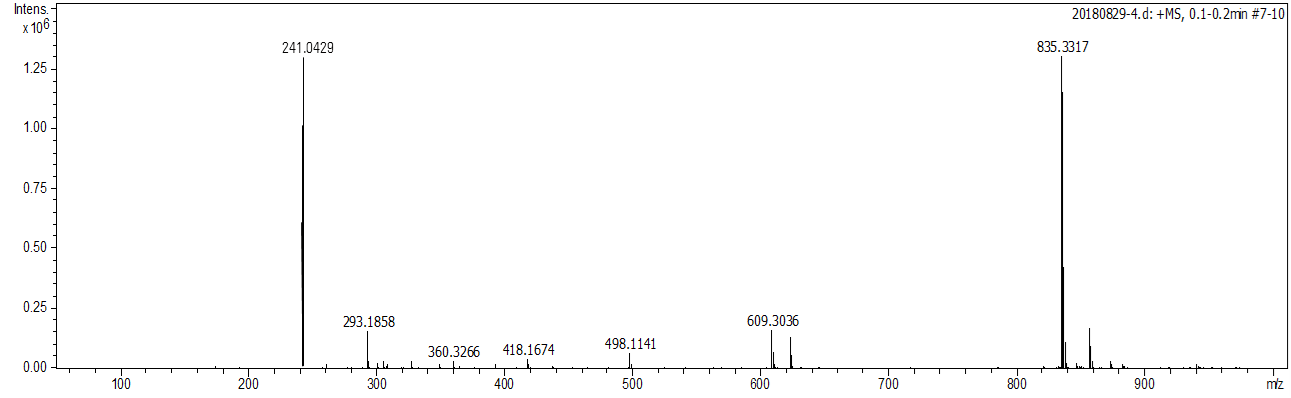


Compound **6e**


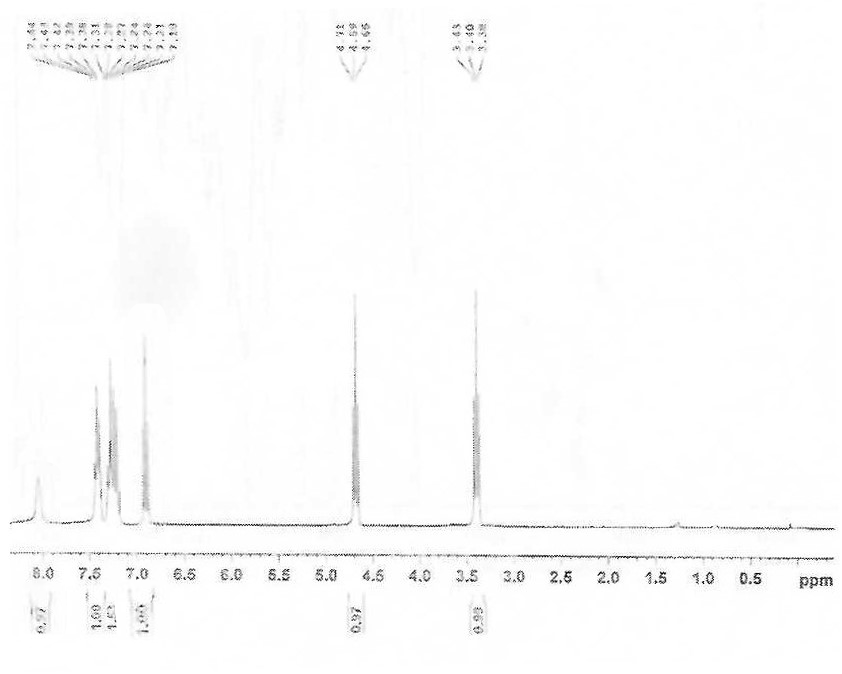


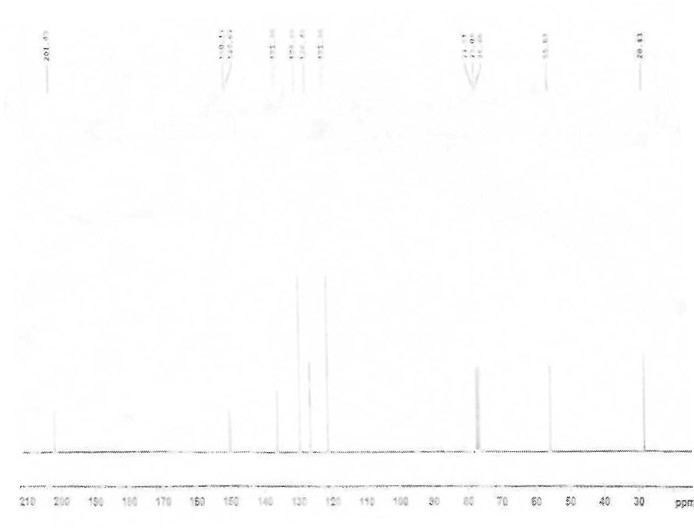


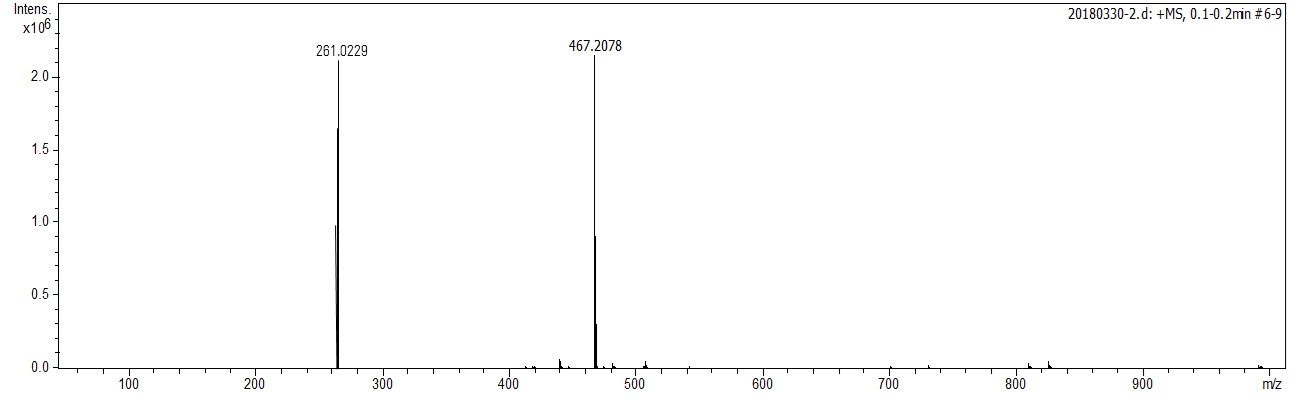


Compound **6f**


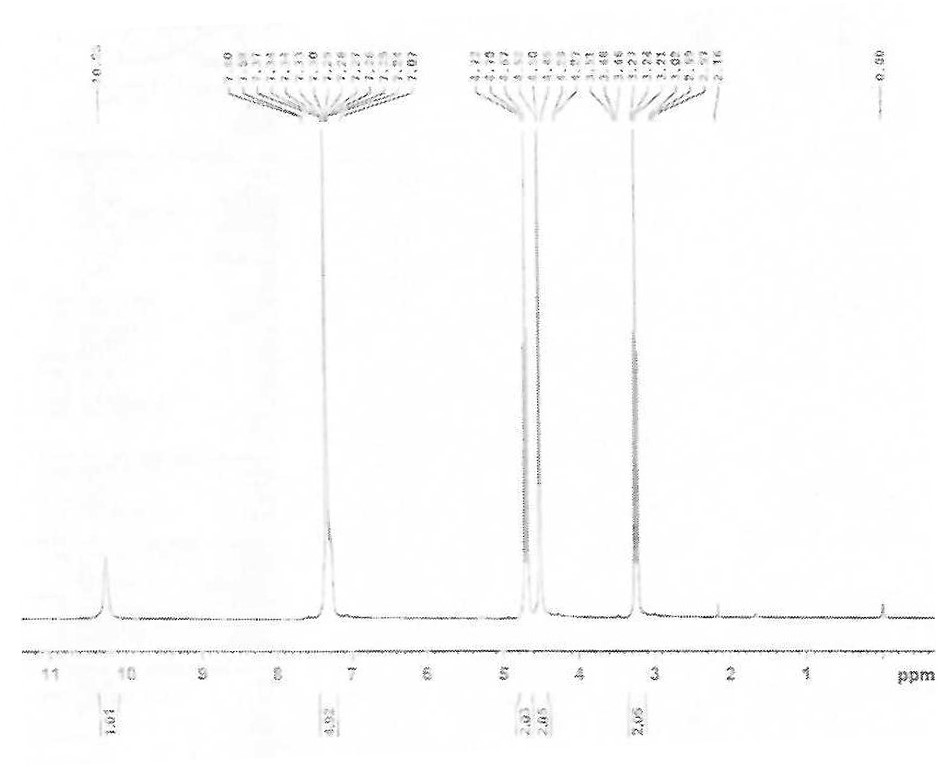


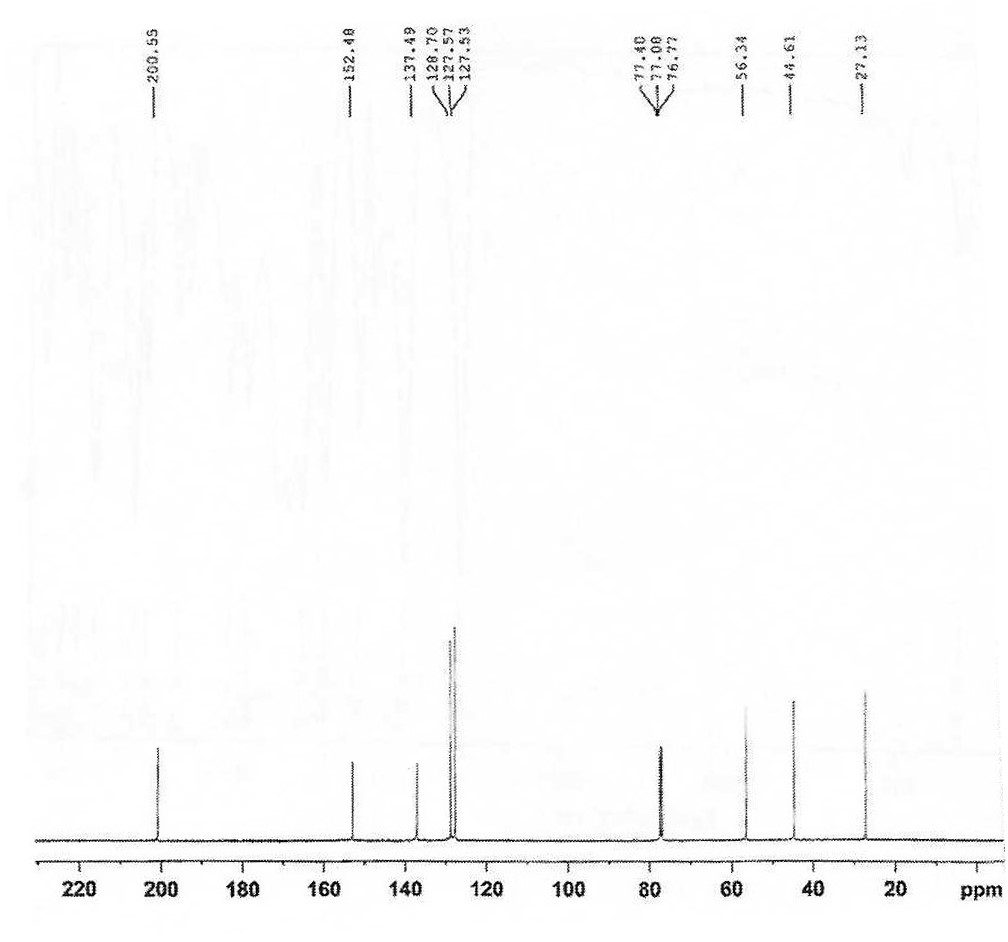


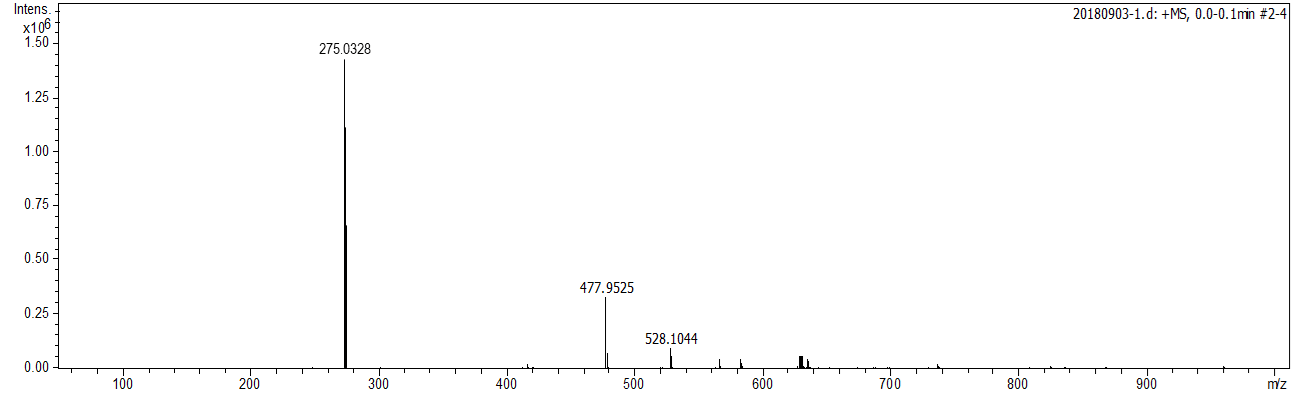


Compound **6g**


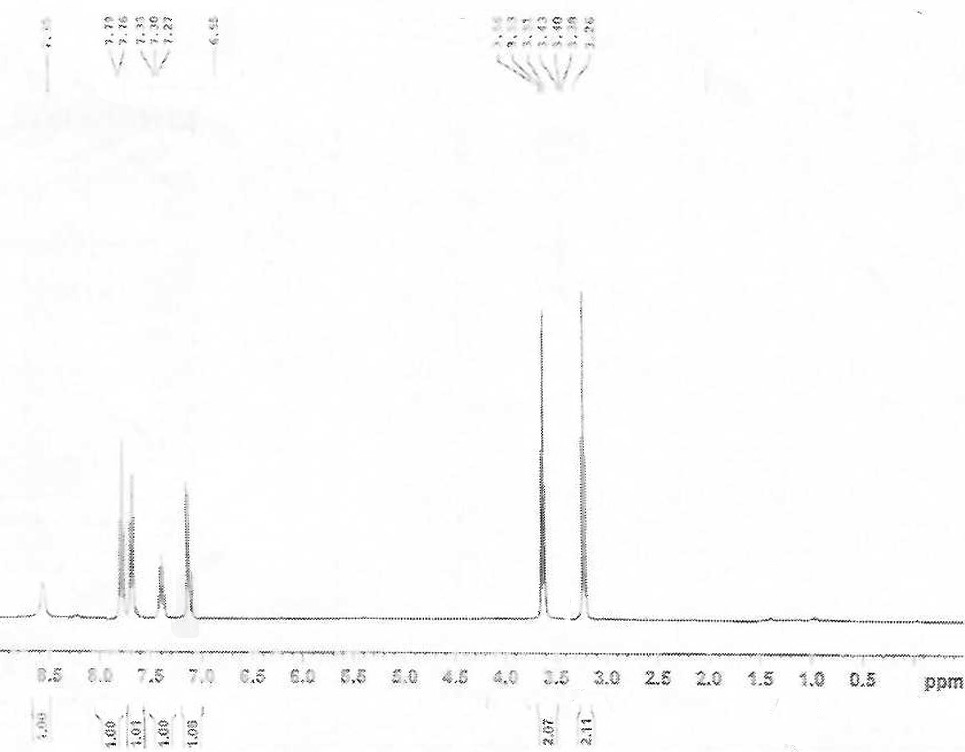


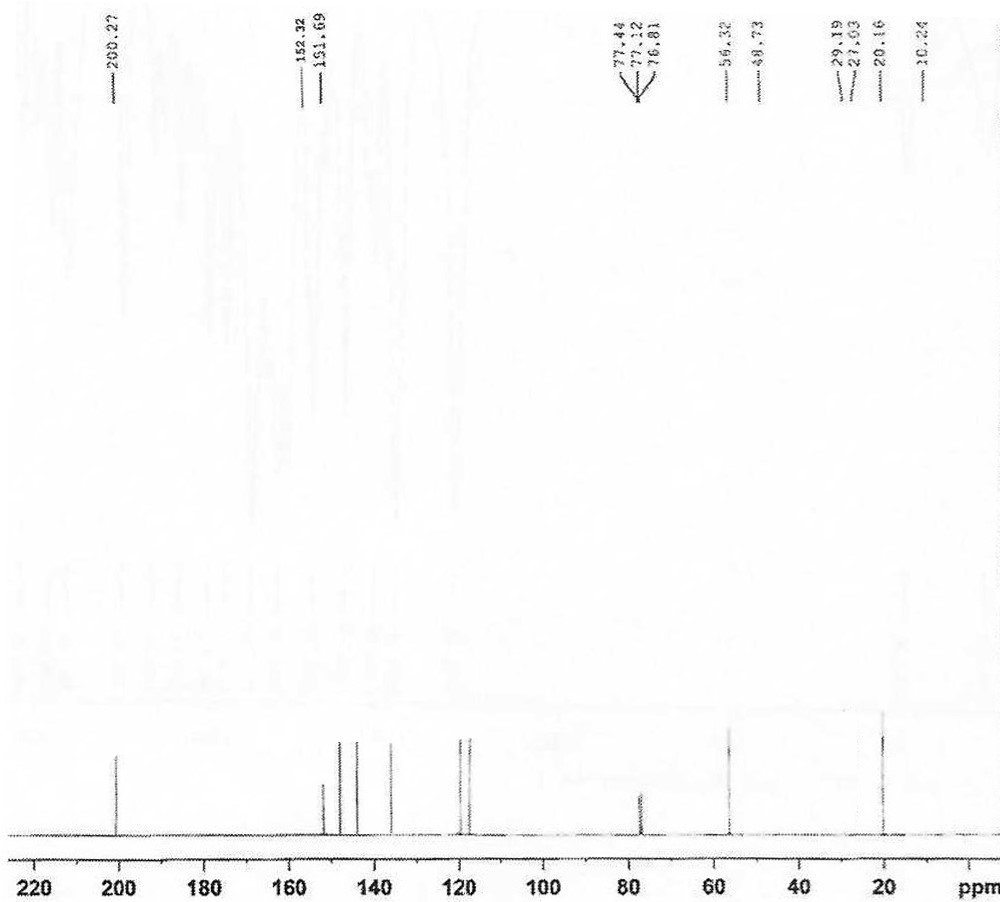


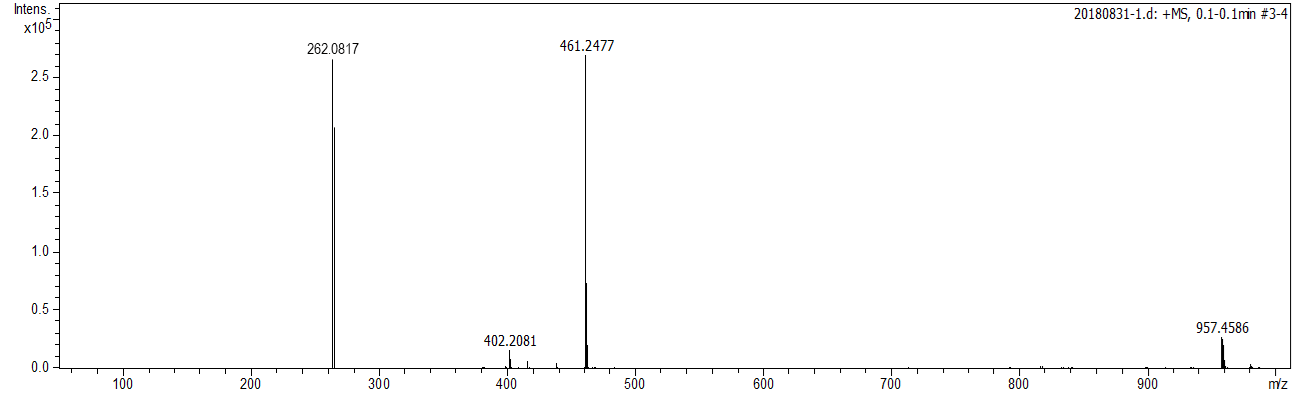


Compound **6h**


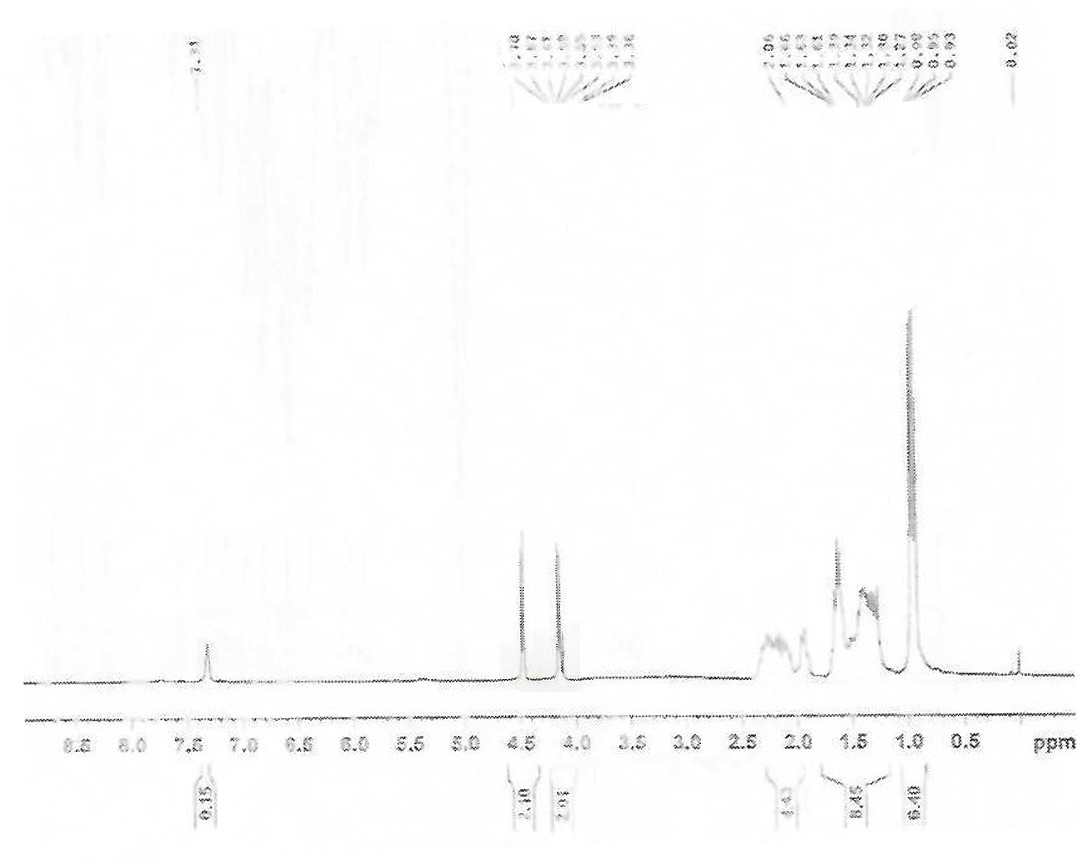


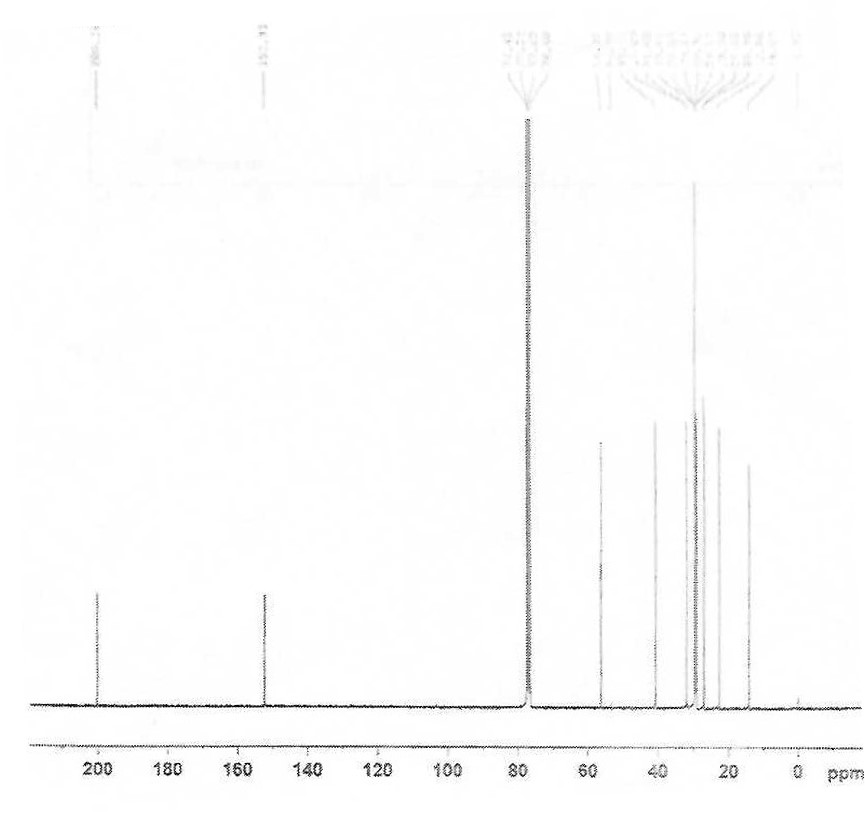


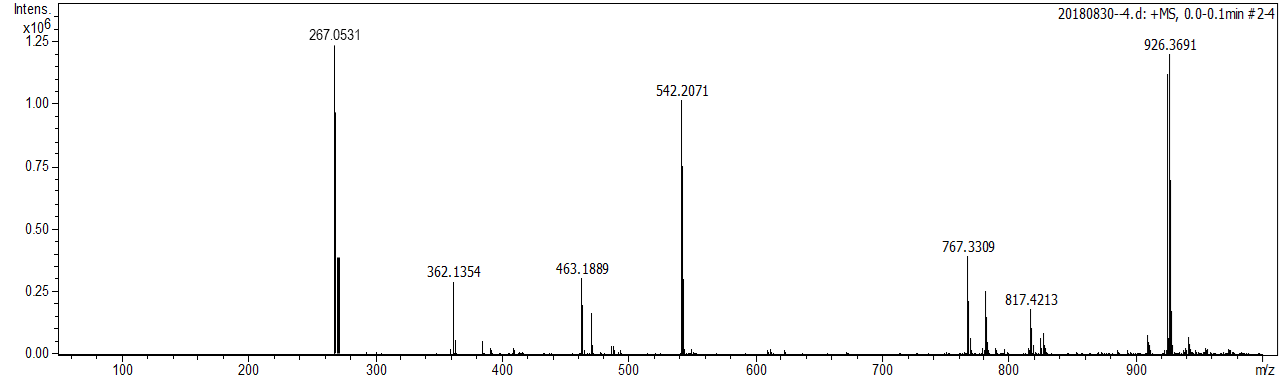


Compound **6i**


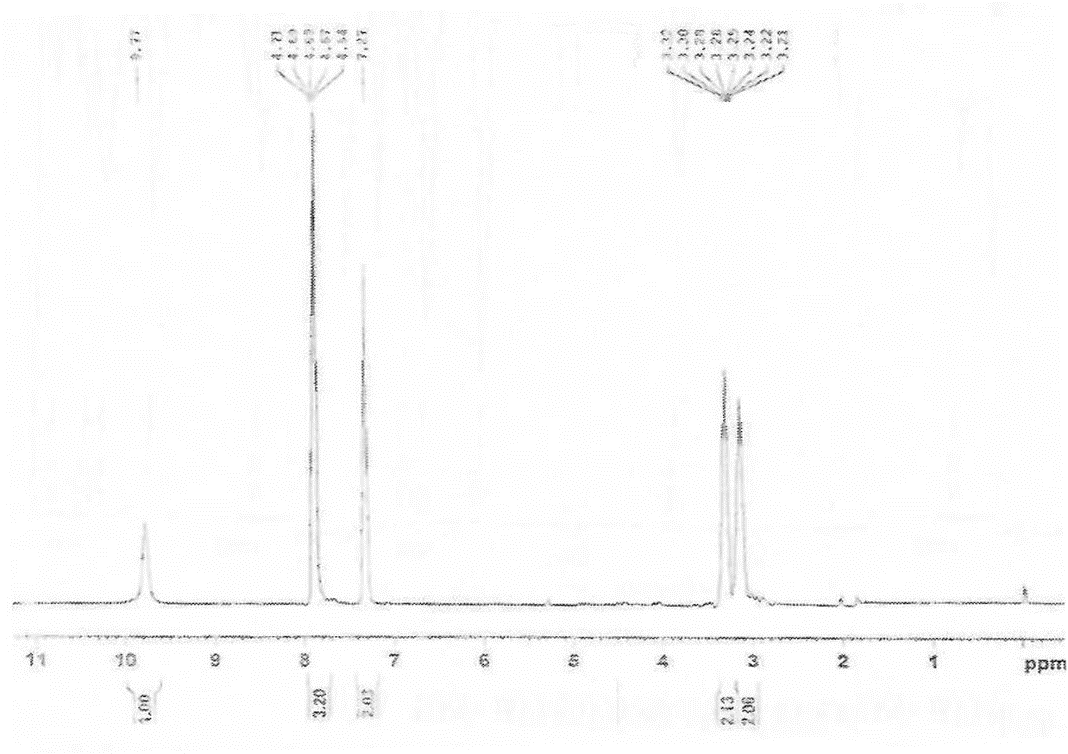


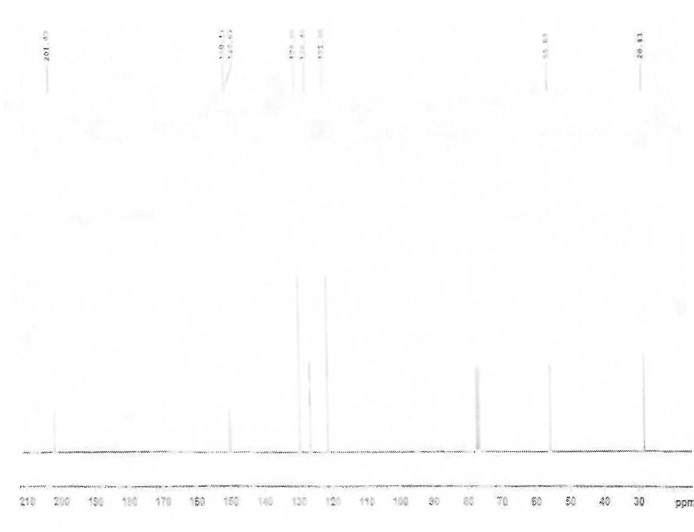


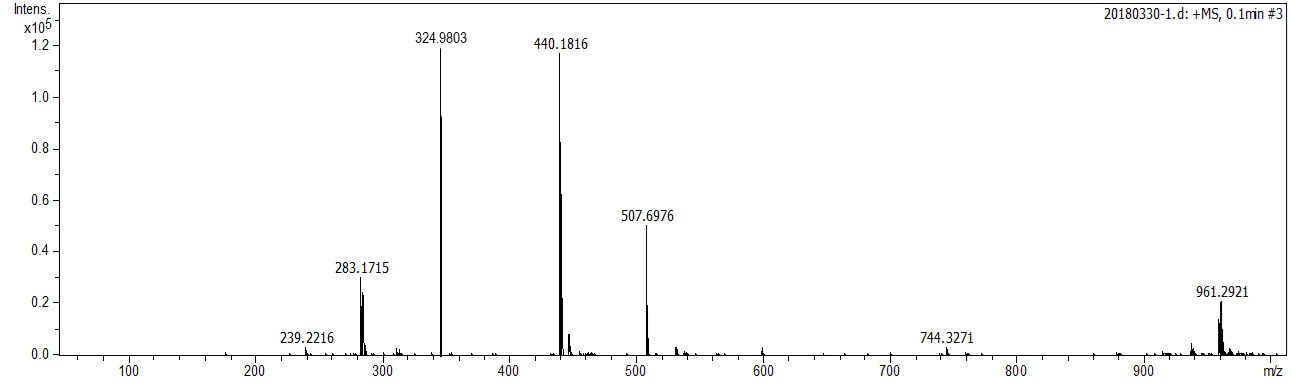


Compound **6j**


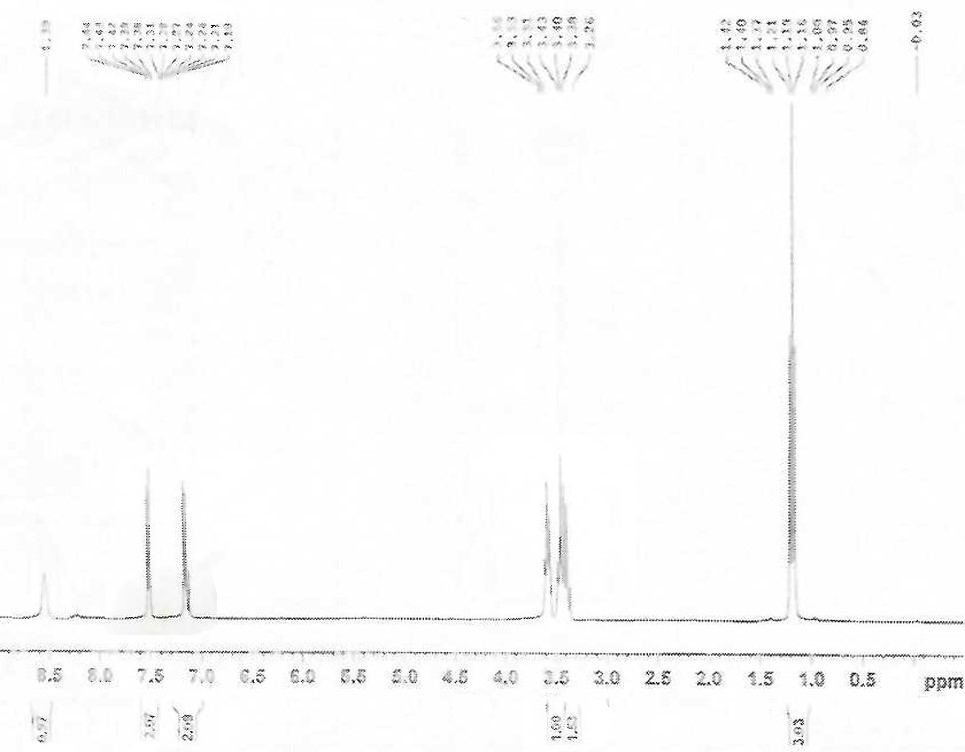


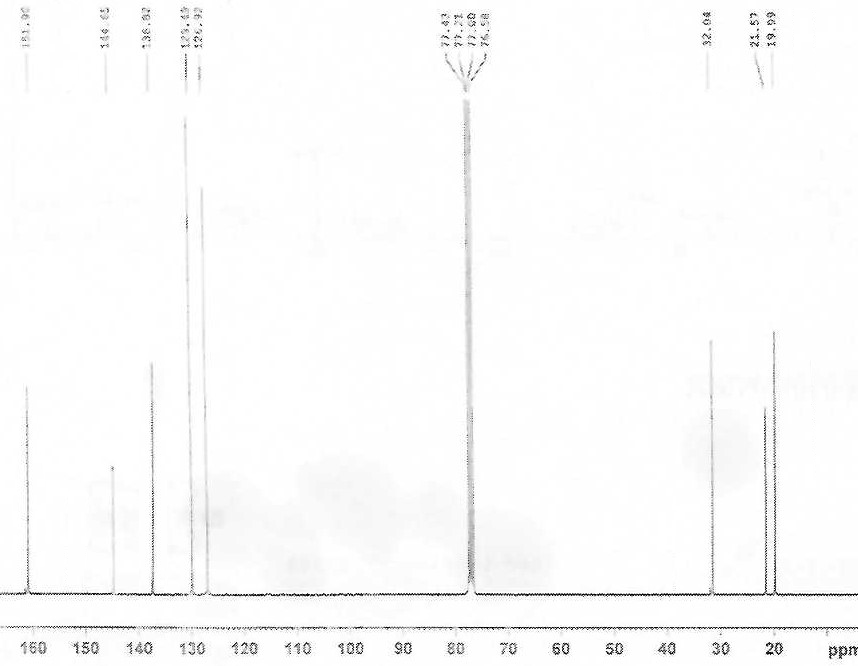


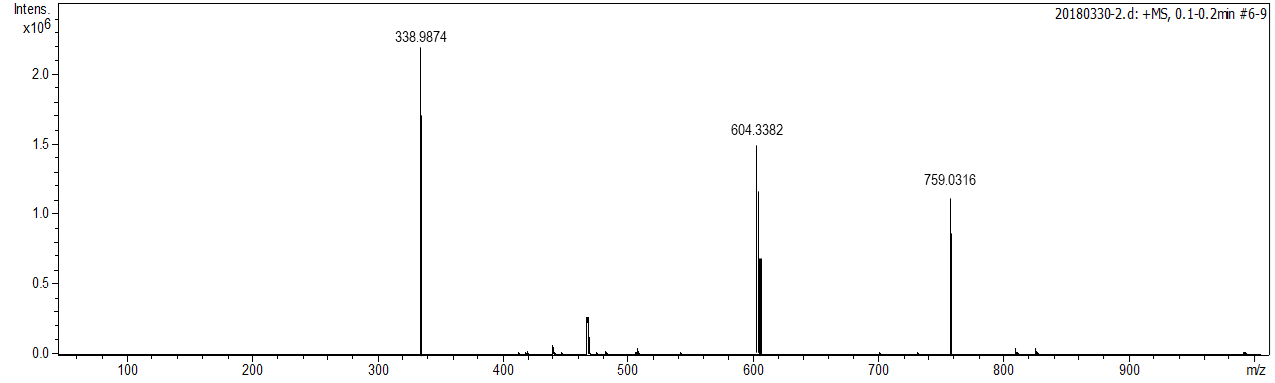


Compound **6k**


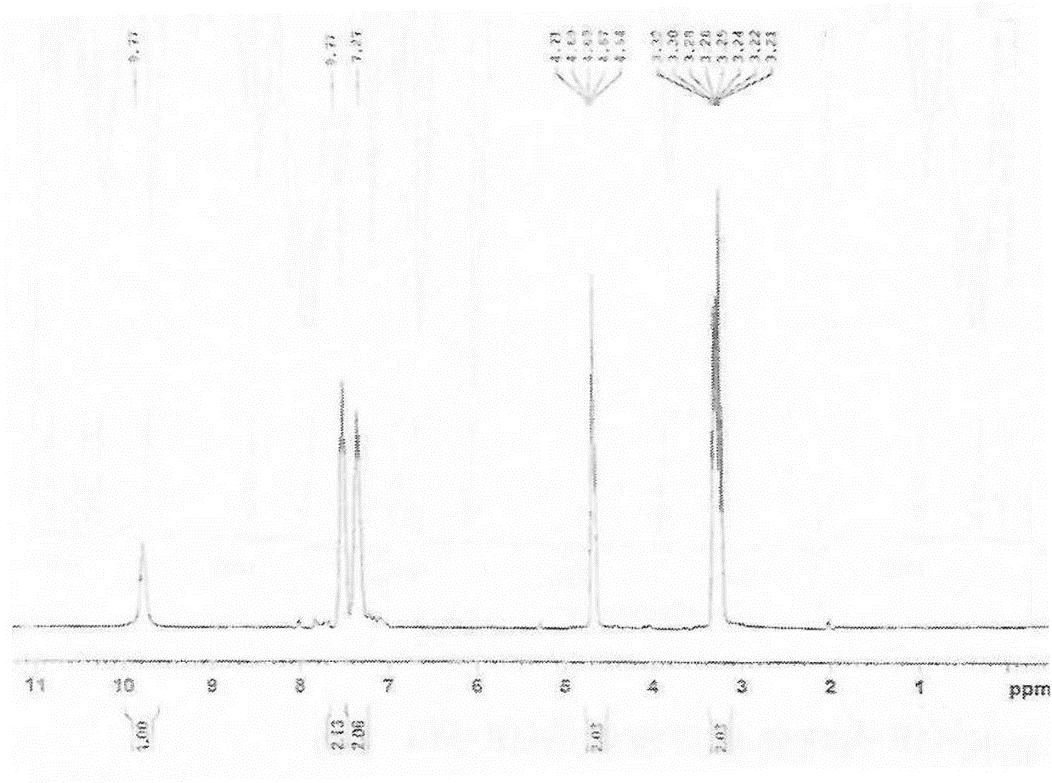


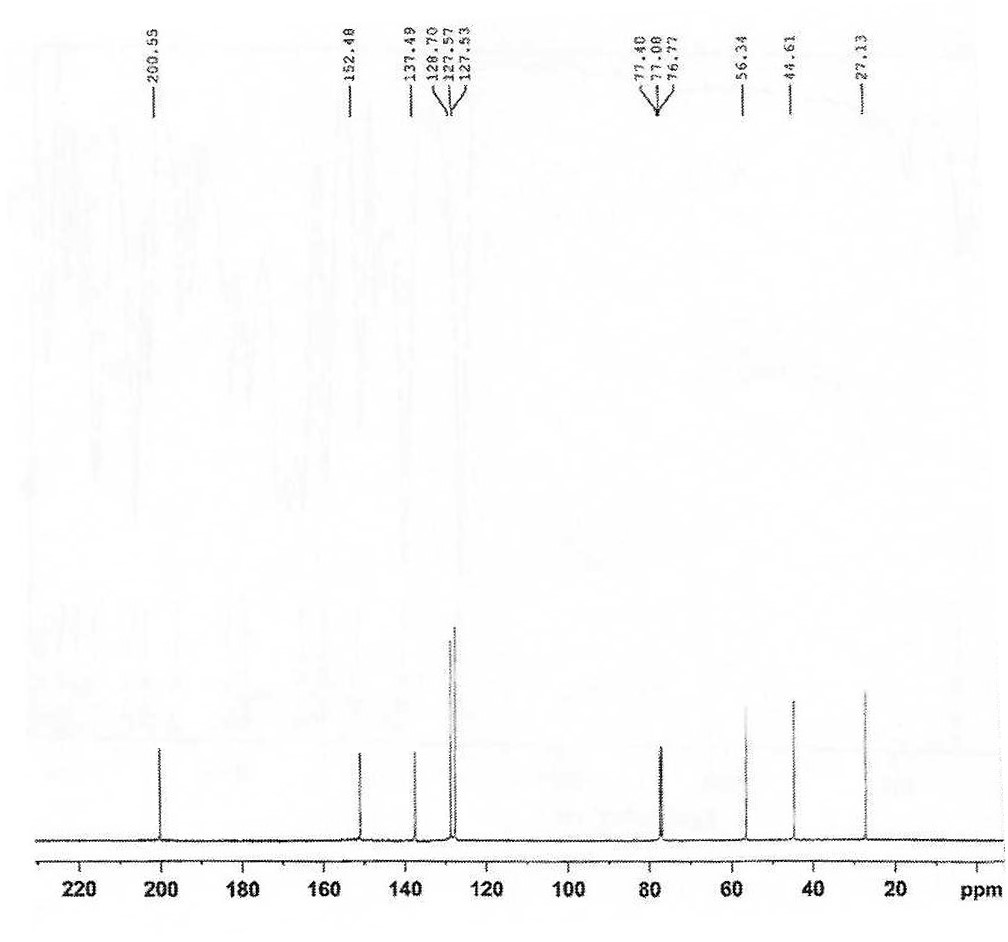


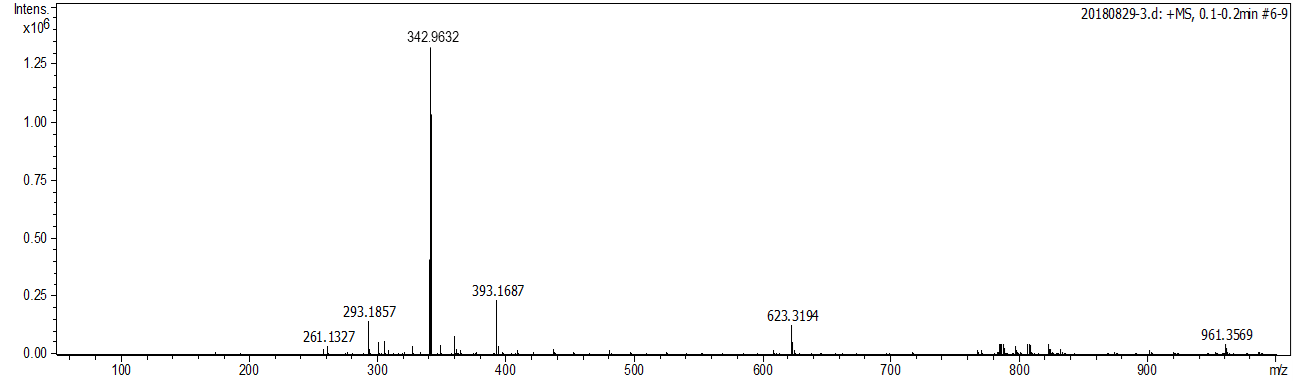


**Minimal underlying data**

**S1 Table.** *In vitro* XO inhibitory potency of thiazolidine-2-thione derivatives.

| Compound | IC_50_ | | | mean | SD |
| --- | --- | --- | --- | --- | --- |
| **3** | 74.13 | 72.25 | 70.07 | 72.15 | 1.66 |
| **4a** | 43.37 | 40.94 | 46.27 | 43.53 | 2.18 |
| **4b** | 50.47 | 57.23 | 66.82 | 58.17 | 6.71 |
| **4c** | 49.37 | 52.16 | 53.28 | 51.60 | 1.64 |
| **4d** | 121.83 | 109.35 | 110.05 | 113.74 | 5.73 |
| **6a** | 19.92 | 23.46 | 25.32 | 22.90 | 2.24 |
| **6b** | 27.15 | 27.62 | 28.64 | 27.80 | 0.63 |
| **6c** | 30.13 | 33.48 | 28.01 | 30.54 | 2.25 |
| **6d** | 21.04 | 17.72 | 18.36 | 19.04 | 1.44 |
| **6e** | 10.56 | 8.38 | 11.67 | 10.20 | 1.37 |
| **6f** | 12.82 | 14.75 | 10.83 | 12.80 | 1.60 |
| **6g** | 17.72 | 15.14 | 10.69 | 14.52 | 2.90 |
| **6h** | 8.25 | 11.08 | 10.29 | 9.87 | 1.19 |
| **6i** | 5.19 | 4.02 | 6.35 | 5.19 | 0.95 |
| **6j** | 11.52 | 8.48 | 9.27 | 9.76 | 1.29 |
| **6k** | 3.19 | 4.42 | 3.06 | 3.56 | 0.61 |
| Allopurinol | 7.74 | 6.81 | 9.04 | 7.86 | 0.91 |
| Febuxostat | 3.02 | 2.91 | 4.08 | 3.34 | 0.53 |

**S2 Table.** Kinetic analysis of compound **6k** inhibited of XO activity.

| Xanthine (mmol/L) | 1/Xanthine | compound **6k** (µmol/L) | | | |
| --- | --- | --- | --- | --- | --- |
|  |  | 0 | 5 | 10 | 20 |
| 2 | 0.5 | 2.73±0.38 | 4.12±0.74 | 4.61±0.49 | 5.36±0.62 |
| 1 | 1 | 3.52±0.66 | 4.94±0.77 | 6.34±0.85 | 7.18±0.49 |
| 0.5 | 2 | 4.64±0.51 | 7.46±1.01 | 11.63±1.38 | 13.63±2.37 |
| 0.25 | 4 | 5.75±1.02 | 16.32±1.84 | 20.47±2.16 | 27.39±1.93 |

| compound **6k** (µmol/L) | Intercept | Slope |
| --- | --- | --- |
| 0 | 1.365 | 1.605 |
| 5 | 1.487 | 3.559 |
| 10 | 2.126 | 4.587 |
| 20 | 2.357 | 6.098 |
